# Tumoral immune microenvironment profiling in B-cell non-Hodgkin lymphoma stratifies patients in different prognostic groups

**DOI:** 10.3389/fimmu.2026.1882562

**Published:** 2026-07-23

**Authors:** Daniel Medina-Gil, Víctor Navarro, Juan C. Nieto, Júlia Carabia, Laura Palomo, Carles Palacio-Garcia, Cristina García, Carlota Pagès-Geli, Pamela Acha, Laura Gallur, Gemma Pujadas, Josep Castellví, Pau Abrisqueta, Francesc Bosch, Marta Crespo

**Affiliations:** 1 Experimental Hematology, Vall d’Hebron Institute of Oncology (VHIO), Instituto de Investigación Sanitaria Hospital Universitari Vall d’Hebron (IIS IR-HUVH), Barcelona, Spain; 2 Department of Medicine, Universitat Autònoma de Barcelona, Bellaterra, Spain; 3 Statistics Unit, Vall d’Hebron Institute of Oncology (VHIO), Instituto de Investigación Sanitaria Hospital Universitari Vall d’Hebron (IIS IR-HUVH), Barcelona, Spain; 4 Servei d’Hematologia, Instituto de Investigación Sanitaria Hospital Universitari Vall d’Hebron (IIS IR-HUVH), Barcelona, Spain; 5 Translational Molecular Pathology, Vall d’Hebron Institute of Research (VHIR), Instituto de Investigación Sanitaria Hospital Universitari Vall d’Hebron (IIS IR-HUVH), Barcelona, Spain

**Keywords:** B-cell lymphoma, immune checkpoints, immune profiling, prognosis, T-cell exhaustion, tumor immune microenvironment

## Abstract

**Background:**

Non-Hodgkin lymphomas (NHL) represent a heterogeneous group of B-cell-derived malignancies. Previous studies showed that tumor immune microenvironment (TIM) components are associated with tumor development and progression. Furthermore, recent transcriptomic analyses revealed that TIM status can stratify patients into distinct prognostic groups; however, these insights are not clinically exploited.

**Methods:**

We characterized the TIM of 64 NHL biopsies and 16 reactive lymphoid tissues (rLT) controls using multi-parameter flow cytometry, including major subpopulations, T-cell subsets and immune checkpoint expression on healthy/malignant B and T cells. Individuals were stratified by TIM profiles, and prognostic values were evaluated. The mutational landscape was also assessed by targeted DNA sequencing.

**Results:**

Flow cytometry analysis revealed HLA-I downregulation in a patient subset and increased expression of inhibitory ligands, particularly in diffuse large B-cell lymphoma (DLBCL). T cells showed a shift toward effector profiles and higher exhaustion marker expression compared with rLT. TIM composition did not differ substantially between patients with specific mutations. TIM-based clustering identified three distinct groups primarily defined by T-cell exhaustion. We assessed the prognostic impact of TIM classification in follicular lymphoma (FL), the most prevalent diagnosis in our cohort. FL patients in cluster C1, characterized by higher frequencies of CD4^+^ and naïve T cells, showed longer time to treatment, overall survival and progression-free survival outcomes compared with FL cases from cluster C2.

**Conclusions:**

The classification of TIM profiles by flow cytometry in NHL patients predicts the outcome of FL patients, highlighting its value for risk stratification and identifying potential immunotherapeutic targets in NHL.

## Introduction

Non-Hodgkin lymphomas (NHL) are a heterogeneous group of lymphoid tumors, with ~85% arising from B-cell clonal expansion (1). Diffuse large B-cell lymphoma (DLBCL) is the most frequent subtype (31%), followed by follicular lymphoma (FL, 22%) and marginal zone lymphoma (MZL, 8%) (2). After first-line treatment, ~30% of DLBCL patients develop relapsed/refractory disease (3), while ~20% of FL patients experience disease progression within 2 years of treatment (4). CAR-T cells and bispecific antibodies have demonstrated improved outcomes by leveraging immune responses (5).

The tumor immune microenvironment (TIM) plays an important role in lymphoma development and progression. *B2M* and *CD58* mutations impair HLA-I and CD58 expression, impeding T-cell responses (6). Interactions between malignant cells and T cells involve multiple immune checkpoint molecules that modulate anti-tumor responses. Lymphoma cells may express co-stimulatory ligands for 41BB(-L), OX40(-L) and ICOS(-L) and upregulate inhibitory ligands for PD-1 (PD-L1) and TIGIT (CD112/CD155) regulating T-cell activation (7, 8). Chronic antigen exposure induces T-cell exhaustion, characterized by sustained expression of inhibitory receptors (PD-1, TIGIT, TIM3, LAG-3, CD244, CD160) and regulatory-T (Treg) cells and Th2-polarization further contribute to immune suppression (9).

TIM characterization involves complex and heterogeneous data, and numerous transcriptomic approaches, mainly from formalin-fixed paraffin-embedded (FFPE) samples, have been developed to stratify lymphoma patients, consistently demonstrating the prognostic value of TIM-based classification (10–15). However, these insights have not yet been translated into routine clinical practice. Here, we hypothesized that flow cytometry-based characterization of the TIM in freshly obtained NHL tumor biopsies, with a particular focus on B-/T-cell crosstalk, could define distinct immune profiles with prognostic significance.

## Materials and methods

### Patient samples

Biopsies from 80 individuals were included in this study, comprising 64 NHL tumor biopsies and 16 reactive and non-malignant lymphoid tissue samples. Patient characteristics are provided in **Supplementary Table S1**. A written informed consent was obtained from all individuals in accordance with the declaration of Helsinki and the study was approved by the local clinical research ethics committee.

### Flow cytometry

Freshly obtained biopsies from lymphoid tissues were mechanically disaggregated and filtered through a 100 µm strainer (BD Biosciences) to obtain a single cell suspension. Cells were stained to assess the frequencies of main lymphocyte subpopulations (CD19, CD3, CD4, CD8, CD56), the expression of inhibitory (PD-L1, CD112, CD155) and co-stimulatory ligands (41BB-L, ICOS-L, OX40-L, CD58) and HLA-I expression in cancer cells (expanded kappa^+^ or lambda^+^ CD19^+^CD20^+^ clone). Additionally, we assessed the expression of inhibitory (PD-1, TIGIT, TIM3, LAG-3, CD244, CD160) and co-stimulatory receptors (41BB, ICOS and OX40) in T cells, as well as T-cell differentiation status, Treg-like and T-helper subpopulations. Detailed information on the flow cytometry panels, fluorochrome-conjugated antibodies, and their concentrations used in this study are provided in **Supplementary Table S2**. 1 x 10^5^ cells were stained in 150 µl of PBS + 0.5% BSA + 0.1% sodium azide at room temperature for 15 minutes per panel. After washing, propidium iodide (BD Biosciences) was added in the tubes specified in **Supplementary Table S2** to exclude dead cells. Samples were acquired in a Navios EX Flow Cytometer (Beckman Coulter) and data was analyzed using FlowJo Software (BD Biosciences).

### Targeted DNA sequencing

Genomic DNA was isolated using DNA Sample Preparation Kit (Roche Diagnostics) and quantified using Qubit dsDNA BR Assay Kit (Thermo Fisher Scientific). DNA was sequenced with a custom NGS panel targeting codifying regions of 190 genes commonly mutated in hematological malignancies (**Supplementary Table S3**). Libraries were prepared from 100 ng of genomic DNA with the KAPA HyperPlus Kit chemistry and captured with KAPA HyperChoice probes (Roche Diagnostics), following the manufacturer’s recommendations. Libraries were sequenced at Centre Nacional d’Anàlisi Genomic (CNAG, Barcelona, Spain) on a NovaSeq 6000 (Illumina) instrument, with a 2 × 150 bp paired-end standard protocol at a mean depth of coverage of 2000x, to detect variants with a variant allele frequency (VAF) down to 1%.

Data analysis was performed using the sarek pipeline version 3.0.2 from nf-co.re project using the human genome build 19 (hg19) (16, 17). The output was processed using an in-house script and variant annotation was performed using ANNOVAR (18). High-probability oncogenic mutations were called by eliminating sequencing and mapping errors, and by filtering out variants with a minor allele frequency > 0.01, according to gnomAD, as well as synonymous variants. Visual analysis with Integrative Genomics Viewer was performed for all cases.

### Statistical analysis

Numerical variable comparisons across different samples were conducted using the Mann Whitney U test. Fisher’s exact test was performed to analyze two dichotomic variables.

For cluster analysis, the k-means method with Euclidean distance was applied. Clusters were visualized in two dimensions using Principal Component Analysis (PCA). The optimal number of clusters was determined using the elbow method, resulting in three clusters. To assess differences between clusters across various variables, the Kruskal-Wallis test was used for numerical variables, while the chi-square test was applied to categorical variables.

For survival analysis, the Kaplan-Meier method was used to estimate survival probabilities, and differences between clusters were evaluated using the log-rank test. Additionally, the hazard ratio (HR) with its confidence interval and p-value was estimated using the Cox proportional hazards model. Multivariable Cox proportional hazard models were employed to identify differences in survival endpoints.

All statistical analyses were performed using GraphPad Prism version 8.0 and R software version 4.3.1. Results were considered statistically significant if p < 0.05.

## Results

### Patient characteristics

**Figure 1:**
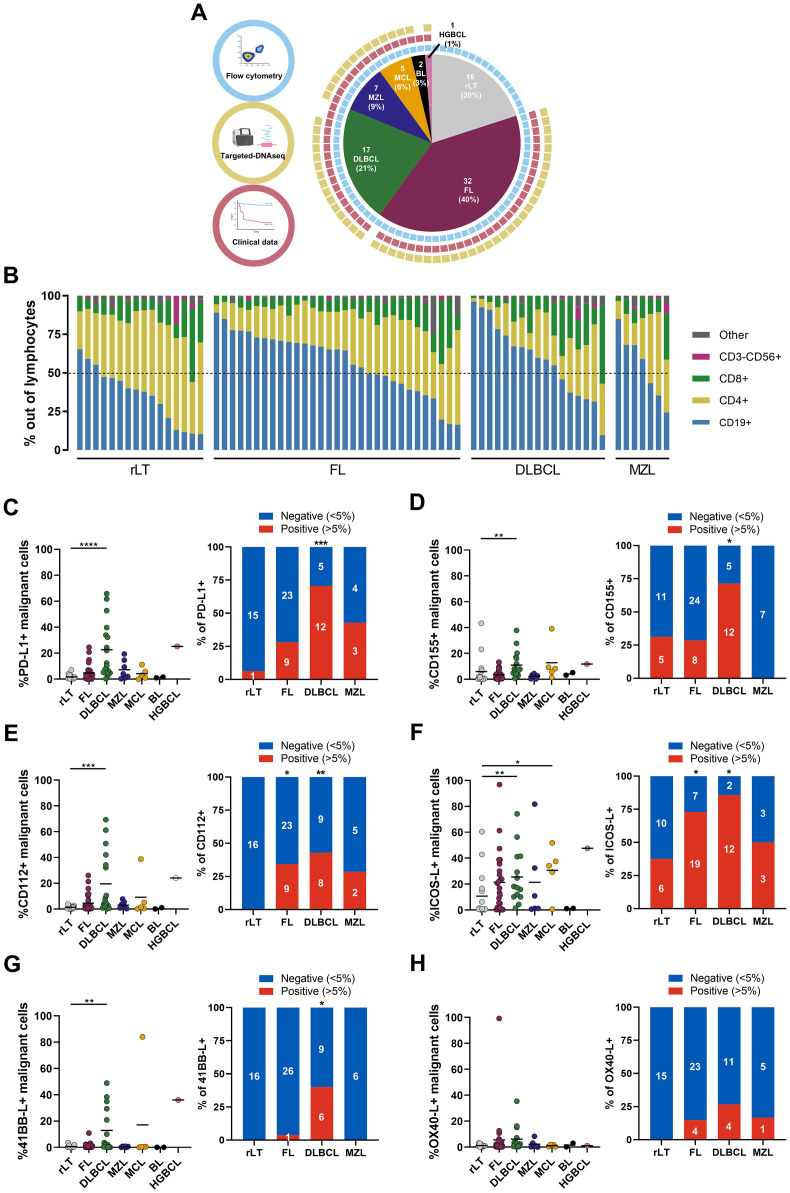
Immune composition and checkpoint ligand expression on healthy and malignant B cells. **(A)** Overview of the study. **(B)** Relative frequency of the main subpopulations of lymphocytes of rLT, FL, DLBCL and MZL. **(C–H)** Percentage of PD-L1^+^, CD155^+^, CD112^+^, ICOS-L^+^, 41BB-L^+^ and OX40-L^+^ malignant cells and healthy B cells within the sample and the number of positive cases (>5% positive B cells) within the diagnostic group. The Mann-Whitney test was performed to compare statistical differences between rLT and NHL subtypes when comparing percentages. Graphs show individual values and mean. Fisher’s exact test was performed to analyze binary variables. (*P<0.05; **P<0.01; ***P<0.001; ****P<0.0001). Reactive lymphoid tissue (rLT), follicular lymphoma (FL), diffuse large B-cell lymphoma (DLBCL), marginal zone lymphoma (MZL), mantle cell lymphoma (MCL), Burkitt lymphoma (BL) and high-grade B-cell lymphoma (HGBCL).

Freshly obtained biopsies from 64 NHL patients were analyzed, comprising 32 FL, 17 DLBCL, 7 MZL, 5 mantle cell lymphomas (MCL), 2 Burkitt lymphomas (BL) and 1 high-grade B-cell lymphoma (HGBCL); additionally, 16 reactive lymphoid tissues (rLT) as controls were immunophenotyped (**Figure 1A**, **S1**; **Supplementary Tables S1, S2**). 67% of tumor samples were collected at diagnosis. Targeted DNA sequencing was performed from FFPE biopsies from 60/64 patients (**Figure 1A**).

Among the 31 sequenced FL samples (97%), *CREBBP* (71%), *KMT2D* (58%) and *TNFRSF14* (39%) were the most frequently mutated genes, followed by *ARID1A* (26%) and *STAT6* (26%). *BCL2* recombination (*BCL2*-R) was detected in 23 out of 30 FL patients (77%) assessed by fluorescence *in situ* hybridization (FISH). In DLBCL, targeted-DNAseq data was available for 16 out of 17 patients (94%), with *KMT2D* (40%) and *CREBBP* (33%) being the most recurrently mutated genes, followed by *MYD88*, *BTG1* and *TP53* each present in 27% of the DLBCL cases. The 7 MZL exhibited more genetic heterogeneity, with *CIITA*, *B2M*, *TNFRSF14*, *KLF2*, *KLHL6*, *TNFAIP3*, *KMT2D* and *TP53* being the most frequently mutated genes (**Supplementary Figures S2A–C**).

### Lymphoma cells express checkpoint ligands more frequently than healthy B cells from rLT

TIM characterization confirmed B cells as the predominant lymphocyte population in lymphomas (**Figure 1B**, **S3A**). A consistently lower frequency of CD4⁺ T cells was observed across all lymphoma subtypes compared with rLT. The proportion of CD8^+^ T cells was similar to the one observed in rLT, leading to an inverted CD4/CD8 ratio which was more pronounced in DLBCL. NK cells were also lower in lymphoma samples, although their percentages were already low in non-malignant tissues (**Supplementary Figures S3B–E**). These data showed a relative expansion of CD8^+^ T cells, as part of the anti-tumor response to the proliferation of neoplastic B cells within the TIM.

Next, HLA-I expression and inhibitory and co-stimulatory ligands on tumor cells (CD19^+^CD20^+^kappa^+^/lambda^+^) were analyzed. The expression of HLA-I was heterogeneous, with several patients exhibiting a lower percentage of HLA-I^hi^ cells, while others showed a high HLA-I^neg^ proportion (**Supplementary Figures S4A–C**). PD-L1 and CD155 expressions were increased in DLBCL, and CD112 was higher in FL and DLBCL compared to rLT. ICOS-L was more frequently expressed in FL and DLBCL, whereas 41BB-L was upregulated only in DLBCL. No differences were observed in OX40-L expression (**Figures 1C–H**; **S5**).

*CD58* mutations, a reported immune evasion mechanism in DLBCL (6), were not detected and CD58 expression was higher in lymphoma than in healthy B cells (**Supplementary Figures S6A–C**), suggesting that while CD2-CD58 immune recognition axis remains intact, lymphoma cells may develop alternative immune evasion mechanisms. Overall, malignant cells expressed more checkpoint ligands than healthy B cells.

### T cells within NHL tumors are mostly effector cells with increased expression of exhaustion markers

**Figure 2:**
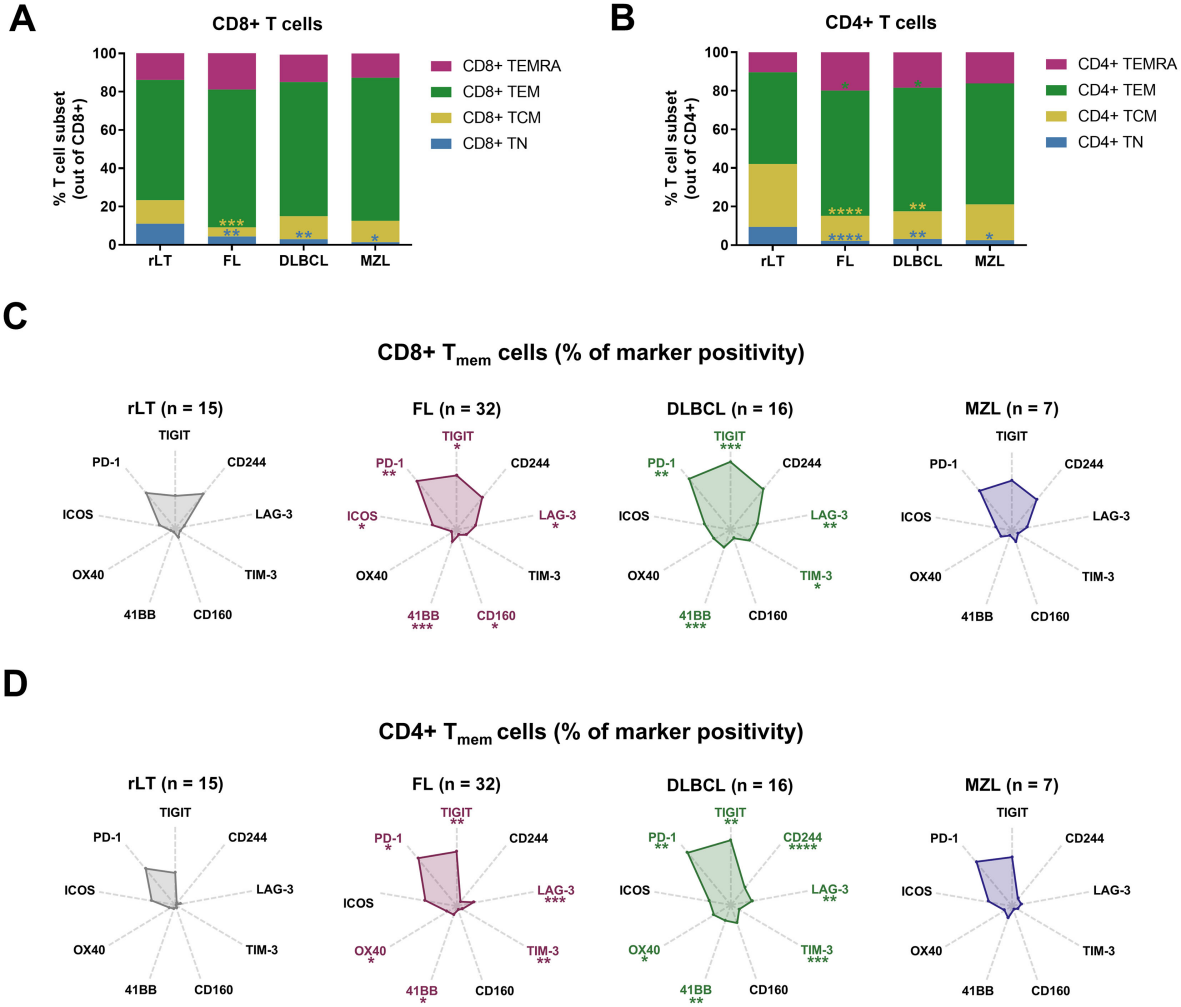
T-cell status in B-cell NHL compared to rLT. **(A)** Stacked bar plot of T-cell differentiation status subtypes including Naïve (CCR7^+^CD45RA^+^), TCM (CCR7^+^CD45RA^-^), TEM (CCR7^-^CD45RA^-^) and TEMRA (CCR7^-^CD45RA^+^) of CD8^+^ T cells and **(B)** CD4^+^ T cells. **(C)** Radar chart of the expression profile of immune checkpoint receptor on CD8^+^ T_mem_ cells, represented as the percentage of marker-positive cells. **(D)** Radar chart of the expression profile of immune checkpoint receptor on CD4^+^ T_mem_ cells, represented as the percentage of marker-positive cells. Lines represent the range of 0% to 100% and dot the mean expression of all the samples within the group. The Mann-Whitney test was performed to compare statistical differences between rLT and NHL subtypes when comparing percentages. (*P<0.05; **P<0.01; ***P<0.001; ****P<0.0001). Reactive lymphoid tissue (rLT), follicular lymphoma (FL), diffuse large B-cell lymphoma (DLBCL), marginal zone lymphoma (MZL), T naïve (TN), T central memory (TCM), T effector memory (TEM), T effector memory re-expressing CD45RA (TEMRA).

T-cell profiling showed a polarization toward effector phenotypes, with effector-memory T cells (TEM) being predominant. FL and DLBCL patients exhibited lower percentages of naïve (TN) CD8^+^ and CD4^+^ T cells and central-memory (TCM) CD4^+^ T cells compared to rLT (**Figures 2A, B**; **S7A–B**), which may indicate a tumor-specific T-cell expansion within NHL niches.

Checkpoint receptor expression was calculated on memory-T (T_mem_) cells, following the TN exclusion (**Figures 2C, D**). In FL, CD8^+^ and CD4^+^ T_mem_ cells showed elevated PD-1, TIGIT and LAG-3 expressions compared to rLT. FL CD8^+^ T_mem_ exhibited decreased CD160, while CD4^+^ T_mem_ expressed more TIM3. DLBCL T_mem_ cells exhibited higher PD-1, TIGIT, LAG-3 and TIM3 expression (**Figures 2C, D**; **S8A–F**, **S9A–F**). DLBCL CD4^+^ T_mem_ cells also showed higher CD244 expression. PD-1 and TIGIT co-expression was more frequent in NHL than in rLT (**Supplementary Figure S8G**, **S9G**). 41BB was upregulated in FL and DLBCL CD8^+^ and CD4^+^ T_mem_ cells. FL CD8^+^ T_mem_ showed increased ICOS expression, whereas FL and DLBCL CD4^+^ T_mem_ cells exhibited significantly higher OX40 expression (**Figures 2C, D**; **S8H–J**, **S9H–J**). PD-1^high^ T_mem_ were more frequent in NHL, reflecting strong exhaustion, and the increased PD-1 expression was mainly observed in effector profiles (**Supplementary Figures S10A–D**). Overall, our data revealed an elevated exhaustion marker expression on antigen-experienced T cells in NHL (**Supplementary Figure S11**).

The frequency of the immunosuppressive subpopulation of Treg-like cells (CD4^+^CD25^+^CD127^low/-^) was elevated in FL and DLBCL compared to controls, whereas Th1, Th2 and Th17 subsets showed no differences (**Supplementary Figures S12A, B**). The comparison between FL and DLBCL revealed higher inhibitory ligand expression on tumor cells and elevated T-cell exhaustion in DLBCL (**Supplementary Table S4**).

Correlation analyses showed an association between the expression of inhibitory-checkpoint ligands in B cells, reflecting tumor immunosuppression. In both CD8^+^ and CD4^+^ T cells, a robust correlation was observed among the expression of the different inhibitory checkpoint receptors analyzed (**Supplementary Figures S13A–C**). PD-1^high^ T cells positively correlated with the expression of other inhibitory receptors, consistent with an exhaustion profile, whereas PD-1^neg^ T cells showed negative correlations with these markers, consistent with a more effector-like phenotype. A correlation matrix integrating all analyzed features revealed a significant correlation between CD8^+^ and CD4^+^ T-cell exhaustion marker expression (**Supplementary Figure S14**).

### TIM-based clustering stratifies lymphoma patients in three groups based on T-cell exhaustion

Next, we aimed to identify common patterns across individuals and classify patients based on TIM characteristics obtained by flow cytometry. For this analysis, we used 54 different parameters obtained from the immunophenotyping, including major lymphocyte subpopulations, differentiated T-cell subsets and expression of inhibitory and costimulatory molecules on healthy/malignant B cells and T cells (**Supplementary Table S5**).

**Figure 3:**
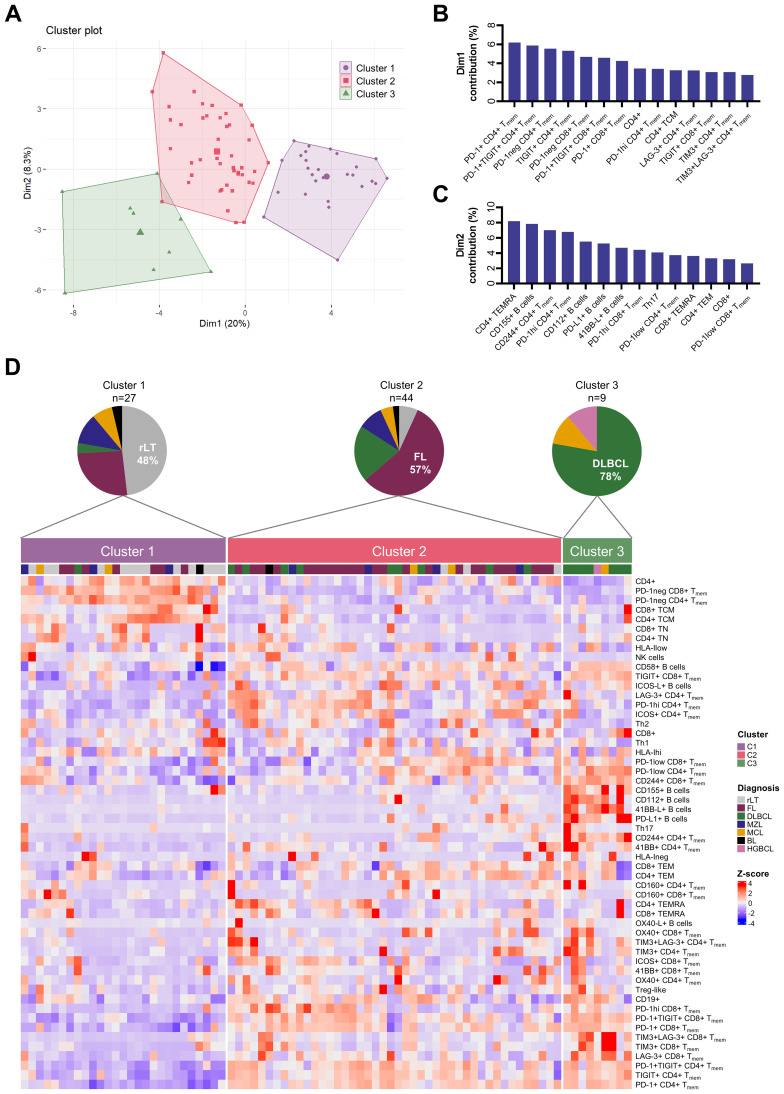
TIM-based clustering analysis of the study cohort. **(A)** Distribution of the different NHL and rLT samples in a PCA plot based on 54 subpopulation frequencies and marker expression. Clusters established by k-means method. **(B)** Contributions to Dim1 and **(C)** Dim2 from the PCA. **(D)** Unsupervised clustered heatmap based on 54 parameters with pie charts depicting the composition of individuals across the k-means-based clusters (n= 80). Non-Hodgkin lymphoma (NHL), Principal Component Analysis (PCA), memory T cells (T_mem_), reactive lymphoid tissue (rLT), follicular lymphoma (FL), diffuse large B-cell lymphoma (DLBCL), marginal zone lymphoma (MZL), mantle cell lymphoma (MCL), Burkitt lymphoma (BL) and high-grade B-cell lymphoma (HGBCL).

By performing unsupervised clustering of the samples using the k-means method, three distinct clusters were identified (**Figure 3A**). The clusters were primarily differentiated by T-cell exhaustion, with PD-1 and TIGIT expression on T_mem_ cells as top contributors of dimension 1 (Dim1). Dimension 2 (Dim2) was defined by CD155, CD112 and PD-L1 expression on B cells, CD4^+^ TEMRA levels, and PD-1^+^ and CD244^+^ CD4^+^ T_mem_-cell levels (**Figures 3B, C**; **Supplementary Table S6**). Cluster 1 (C1) (n=27) consisted of 48% of rLT, 26% FL, 11% MZL, 7% MCL, 4% DLBCL and 4% BL. Cluster 2 (C2) (n=44) consisted of 57% FL, 20% DLBCL, 9% MZL, 7% rLT, 5% MCL and 2% BL. Cluster 3 (C3) (n=9) consisted of 78% DLBCL, 11% MCL and 11% HGBCL. rLT mainly clustered on C1 (81%), a group enriched in CD4^+^ T cells, PD-1^neg^ expression and elevated TCM and TN cell proportions. Most FL cases grouped in C2 (78%), a cluster characterized by higher PD-1, TIGIT, ICOS and LAG-3 expression on T_mem_. Most DLBCL cases clustered in C3 (41%) and characterized by a higher inhibitory ligand expression and increased TIM3^+^ T_mem_ cells (**Figure 3D**).

Next, we evaluated whether TIM-based clustering was associated with specific genetic alterations. In our cohort, no significant enrichment of genetic mutations was observed within any of the clusters. Moreover, despite minor differences in a few parameters, the overall TIM composition did not differ substantially between patients with or without specific mutations (**Supplementary Figure S15**), suggesting that while genetic alterations contribute to shaping the TIM, they do not fully determine its composition.

### TIM-based clustering stratifies FL patients in two different prognostic groups

**Figure 4:**
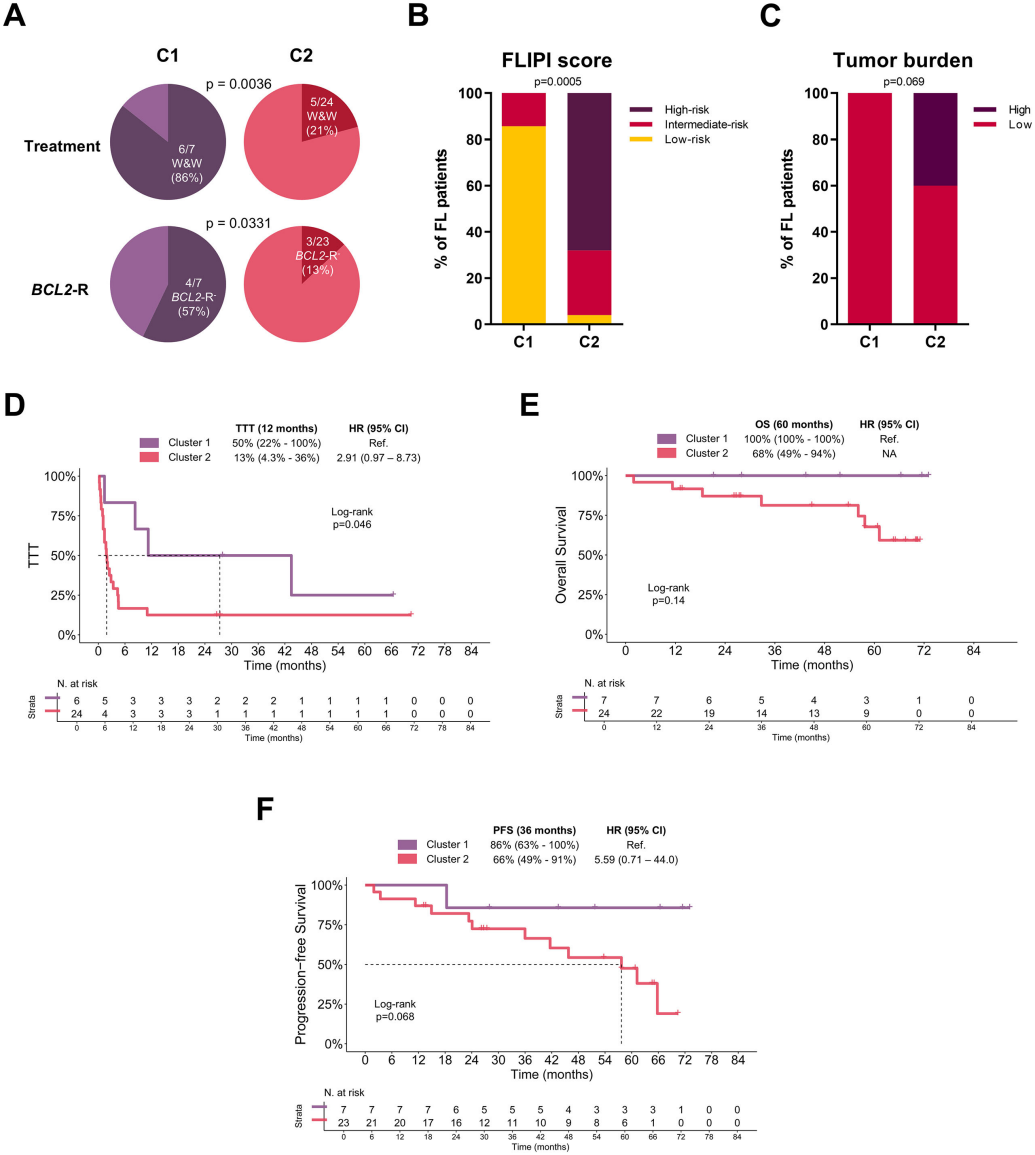
Impact of TIM-based clustering on FL prognosis. **(A)** Distribution of the FL patients based on TIM clusters and treatment management and the presence or not of *BCL2*-R. **(B)** Comparison of the FLIPI score between patients from C1 and C2. **(C)** Comparison of the tumor burden between patients from C1 and C2 based on TIM clustering. **(D)** Comparison of the TTT between C1 and C2 defined as the time from the sample to the next treatment. **(E)** Comparison of the OS between C1 and C2 from the sample. **(F)** Comparison of the PFS between C1 and C2 from the sample to the relapse after the next treatment. Cluster 1 (C1), Cluster 2 (C2), Follicular Lymphoma International Prognostic Index (FLIPI), time to treatment (TTT), overall survival (OS), progression-free survival (PFS), hazard ratio (HR), confidence interval (CI).

To evaluate whether TIM-based clusters are associated with clinical features, we focused on FL, the most common diagnosis in our cohort. FL cases in C1 (n=7), characterized by a reactive phenotype, were compared with those in C2 (n=25), which exhibited a more exhausted T-cell phenotype. C1 was enriched for watch-and-wait (W&W) cases (p=0.0036) and *BCL2*-R-negative FL cases compared to C2 (**Figure 4A**). However, TIM composition did not significantly differ between *BCL2*-R-negative and positive cases (**Supplementary Figure S16**), likely due to clustering of 3 *BCL2*-R-negative cases with *BCL2*-R-positive cases. Significant differences were observed in the Follicular Lymphoma International Prognostic Index (FLIPI) score, with C1 showing predominantly low-risk patients and C2 grouping high-risk cases. Additionally, C2 grouped FL patients with a high tumor burden although most patients exhibited a low burden (**Figures 4B, C**). Most FL patients retained follicular structure, with no differences between clusters (**Supplementary Figure S17A**).

We next evaluated the prognostic value of this classification. Patients from C1 displayed better time to treatment (TTT) outcomes (12-month TTT 50% vs 13%, p=0.046) (**Figure 4D**). Patients in C2 experienced poorer overall survival (OS) compared to C1, although significant differences were not observed (60-month OS 100% vs 68%, p=0.14) (**Figure 4E**). Lastly, FL cases from C1 also had longer progression-free survival (PFS) (36-month PFS 86% vs 66%, p=0.068) compared to C2 (**Figure 4F**). Collectively, these results revealed that TIM-based clustering can stratify FL patients in different prognostic groups.

Multivariate analyses showed that the prognostic impact of TIM-based clustering is influenced by FLIPI score and tumor burden (**Supplementary Figures S17B, C**), suggesting that TIM status may provide additional immunological context to enrich current risk stratification models. Collectively, our findings highlight the utility of TIM immune profiling in lymphoma patients for prognostic stratification and for identifying potential immunotherapeutic targets to enhance T-cell mediated anti-tumor responses.

## Discussion

Deciphering the architecture of the tumor microenvironment in NHL has been a major research focus in recent years due to its critical role in disease development, progression, and treatment resistance. Recently, efforts have been made to stratify DLBCL patients into different prognostic groups based on TIM characteristics derived from RNA sequencing. However, these approaches are not exploited in clinics. In this study, we characterized TIM across NHL subtypes at genetic and immunophenotypic levels and evaluated its potential for patient stratification and prognostic assessment.

Regarding the mutational landscape, *CREBBP*, *KMT2D* and *TNFRSF14* were the most frequently mutated genes in FL, consistent with previous studies (19, 20). DLBCL showed frequent *KMT2D* and *CREBBP* mutations, followed by *BCL10*, *MYD88*, *BTG1*, and *TP53*, also in line with previous reports (21, 22).

Flow cytometry analysis revealed high heterogeneity in HLA-I expression, with several patients exhibiting reduced HLA-I^hi^ expression and few showing a high percentage of HLA-I^neg^ cells. Loss or downregulation of HLA-I has been previously described in various NHL subtypes (6, 23, 24). Additionally, we observed increased expression of inhibitory checkpoint ligands, PD-L1 and CD155 in DLBCL, as well as CD112 in both FL and DLBCL, compared with rLT. A previous study similarly reported higher expression of CD155 and CD112 in DLBCL cases compared to tonsils; however, they did not detect CD112 expression on FL cells (7). In our cohort, PD-L1 expression in DLBCL cells was observed in approximately 70% of patients, consistent with previous reports, which have shown substantial heterogeneity in PD-L1 positivity among DLBCL cases, ranging from 11% to 61% (7, 25–27).

Regarding co-stimulatory molecules, we observed an upregulation of ICOS-L and 41BB-L in lymphoma cells, particularly in DLBCL cells. Increased ICOS-L expression on lymphoma cells compared with B cells from reactive lymphadenitis upon *in vitro* stimulation has previously been reported (28). In addition, immunohistochemical analyses have shown that multiple B-cell NHL subtypes exhibit both surface and intracellular expression of 41BB-L (29).

Although *CD58* mutations impairing surface expression have been described in DLBCL, we did not detect these mutations in our cohort, and CD58 expression remained intact (6). While *CD58* mutations are recurrent in DLBCL, they are not the most frequent (22, 30). Given our small DLBCL cohort size, these mutations may not be represented in our dataset.

T-cell profiling showed a polarization toward effector phenotypes, with a marked reduction in the naïve pools of CD8^+^ and CD4^+^ T cells compared to rLT. Similar findings have been reported when comparing tumor samples with tonsils (7). T-cell exhaustion was evident in both FL and DLBCL, as indicated by increased expression of inhibitory receptors on CD4^+^ and CD8^+^ T cells including PD-1, TIGIT, TIM3, LAG-3, CD244 and CD160, consistent with prior studies (7, 31, 32). NHL also exhibited a polarization toward a higher frequency of PD-1^high^ T cells compared to rLT, which correlated with the expression of other inhibitory receptors. This increase occurred at the expense of PD-1^neg^ subsets, which showed a negative correlation with inhibitory receptor expression and present a more effector-like phenotype, further supporting a more exhausted T-cell phenotype in lymphoma patients. Additionally, 41BB was also expressed on CD4^+^ and CD8^+^ T cells in FL and DLBCL which has also been previously reported (33). Our immunophenotyping also revealed an increased frequency of intratumoral Tregs in FL and DLBCL compared to non-malignant tissues, as previously described (34). The roles of Th1, Th2 and Th17 in NHL remain unclear, and we observed no significant differences in their proportions. Overall, our findings indicate a distinct pattern of T-cell exhaustion, with elevated frequency of Tregs, highlighting potential therapeutic targets such as PD-1 or TIGIT.

DLBCL patients exhibited higher levels of T-cell exhaustion compared to FL and rLT. Bispecific antibodies and CAR-T cell therapies have demonstrated high response rates in B-cell malignancies, and previous reports have shown that the status of T cells in peripheral blood is a determinant of response to these therapies (35–37). However, while exhausted T-cell phenotypes in peripheral blood have been linked to poorer outcomes following T-cell–redirecting therapies, whether similar features within the DLBCL tumor microenvironment influence response remains an open question. TIM comprises a heterogeneous mixture of different cell types and functional states, and numerous approaches have been developed to reduce dimensionality and stratify lymphoma patients based on TIM characteristics. Most of these studies have relied on transcriptomic data, primarily from FFPE tissues (10, 11, 13). Previous mass cytometry analyses characterizing intratumoral CD4^+^ T cell subpopulations also identified specific PD-1^+^ T cell subpopulations associated with poor prognosis (38).

In this study, unsupervised clustering of TIM features from multi-parameter flow cytometry identified three distinct groups with diagnostic heterogeneity and stratified FL patients into two subgroups with distinct immune profiles and clinical outcomes. Notably, C1 was enriched in W&W patients, *BCL2*-R-negative FL cases and was characterized by a less exhausted T-cell phenotype. These findings align with prior transcriptomic studies demonstrating the prognostic relevance of TIM status in FL (10, 12). In particular, terminally exhausted T-cell signatures have been linked to adverse prognosis across different NHL subtypes, including FL (15).

This study was limited by the availability of fresh tumor biopsies and the biological heterogeneity of NHL, which constrained the number of cases available for analysis within lymphoma subtypes. Furthermore, flow cytometry-based approaches cannot capture the spatial TIM architecture or the organization of functional immune synapses within the tumor niche. Therefore, future studies incorporating spatial and transcriptomic approaches applicable to FFPE samples will provide complementary information on TIM organization to improve its predictive and prognostic value. Collectively, this study demonstrates that TIM immunophenotyping of freshly obtained NHL biopsies, generally not assessed in clinical practice, can predict lymphoma patient outcomes and identify immunotherapeutic targets to enhance anti-tumor T-cell responses.

## Data Availability

Raw data (FASTQ files) from the targeted DNA sequencing are deposited in the European Genome-Phenome Archive (EGA) (https://ega-archive.org) under controlled access (study ID: EGAS50000001964; dataset ID: EGAD50000002845). Access is provided upon request for approved biomedical research, subject to ethical approval.
