# Supplementary material for: Tumoral immune microenvironment profiling in B-cell non-Hodgkin lymphoma stratifies patients in different prognostic groups
Source: Front Immunol. 2026 Jul 23;17:1882562. doi: 10.3389/fimmu.2026.1882562 (PMC13442453; doi:10.3389/fimmu.2026.1882562)

SUPPLEMENTAL MATERIAL

SUPPLEMENTAL TABLE LEGENDS

Table S1. Patient characteristics.

Table S2. Flow cytometry panels and the information on the fluorochrome-conjugated antibodies and the concentrations used in the study.

Table S3. Genes included in the NGS panel.

Table S4. Statistical comparison of the tumour immune microenvironment status between FL and DLBCL cases.

Table S5. List of the 54 flow cytometry parameters related to the tumour immune microenvironment included in the Principal Component Analysis (PCA).

Table S6. Principal Component Analysis (PCA) of the immune profiles from rLT and NHL samples (n=80).

SUPPLEMENTAL FIGURE LEGENDS

Supplemental figure S1. Gating strategy for the lymphocyte subpopulations characterized in this study. Representative flow cytometry dot plots illustrating the gating strategy used in each panel to define the lymphocyte subpopulations characterized in this study.

Supplemental figure S2. Next Generation Sequencing (NGS). A. Top recurrently mutated genes in FL (n = 31). **B.** Top recurrently mutated genes from DLBCL samples (n = 15). **C.** Top recurrently mutated genes from MZL samples (n = 8). Follicular lymphoma (FL), diffuse large B-cell lymphoma (DLBCL) and marginal zone lymphoma (MZL)

Supplemental figure S3. Distribution of the main lymphocyte subpopulations in NHL. A. Percentage of CD19+ cells out of lymphocytes. **B.** Percentage of CD8+ T cells out of lymphocytes. **C.** Percentage of CD4+ T cells out of lymphocytes. **D.** Ratio CD4/CD8. **E.** Percentage of NK cells (CD3-CD56+) out of lymphocytes. The Mann-Whitney test was performed to compare statistical differences between rLT and NHL subtypes when comparing percentages. Graphs show individual values and mean. (*P<0.05; **P<0.01; ***P<0.001; ****P<0.0001). Reactive lymphoid tissue (rLT), follicular lymphoma (FL), diffuse large B-cell lymphoma (DLBCL), marginal zone lymphoma (MZL), mantle cell lymphoma (MCL), Burkitt lymphoma (BL) and high-grade B-cell lymphoma (HGBCL).

Supplemental figure S4. HLA-I expression of lymphoma cells. A. Gating strategy of polyclonal B cells from rLT and malignant B cells from NHL samples. **B.** Representative flow cytometry plots of the analysis of the HLA-I expression. **C.** Percentages of HLA-I high, low, and negative expression out of malignant clone ($CD19^+CD20^+$ and κ^+ or λ^+) compared to healthy B cells ($CD19^+CD20^+$). Mann-Whitney test was performed to compare statistical differences between rLT and NHL subtypes. Graphs show individual values and mean. (*P<0.05; **P<0.01; ***P<0.001; ****P<0.0001). Reactive lymphoid tissue (rLT), follicular lymphoma (FL), diffuse large B-cell lymphoma (DLBCL), marginal zone lymphoma (MZL).

Supplemental figure S5. Analysis of inhibitory and co-stimulatory ligands on lymphoma cells. Representative flow cytometry plots of CD19-CD20- cells, B cells from rLT and malignant cells of PD-L1, CD155, CD112, ICOS-L, 41BB-L and OX40-L expression.

Supplemental figure S6. CD58 expression on lymphoma cells. A. Representative flow cytometry plots of CD58 expression on CD19-CD20- cells from a NHL sample, B cells from a rLT sample and malignant cells from a NHL sample. **B.** Percentage of $CD58^+$ healthy/malignant B cells. **C.** MFI of CD58 expression of healthy/malignant B cells. Mann-Whitney test was performed to compare statistical differences between rLT and NHL subtypes when comparing percentages and intensity of expression. Graphs show individual values and mean. (*P<0.05; **P<0.01; ***P<0.001; ****P<0.0001). MFI: mean fluorescence intensity. Reactive lymphoid tissue (rLT), follicular lymphoma (FL), diffuse large B-cell lymphoma (DLBCL), marginal zone lymphoma (MZL), mantle cell lymphoma (MCL), Burkitt lymphoma (BL) and high-grade B-cell lymphoma (HGBCL).

Supplemental figure S7. T cell differentiation status in patients with NHL. A. Representative dot plot of CCR7 and CD45RA expression on CD8+ T cells and the percentage of T cell differentiation status subtypes of $CD8^+$ T cells including TN ($CCR7^+CD45RA^+$), TCM ($CCR7^+$CD45RA-), TEM (CCR7-CD45RA-) and TEMRA (CCR7-$CD45RA^+$). **B.** Representative dot plot of CCR7 and CD45RA expression on CD4+ T cells and the percentage of T cell differentiation status subtypes of $CD4^+$ T cells including Naïve ($CCR7^+CD45RA^+$), TCM ($CCR7^+$CD45RA-), TEM (CCR7-CD45RA-) and TEMRA (CCR7-$CD45RA^+$). The Mann-Whitney test was performed to compare statistical differences between rLT and NHL subtypes when comparing percentages. Graphs show individual values and mean. (*P<0.05; **P<0.01; ***P<0.001; ****P<0.0001). Reactive lymphoid tissue (rLT), follicular lymphoma (FL), diffuse large B-cell lymphoma (DLBCL), marginal zone lymphoma (MZL), mantle cell lymphoma (MCL), Burkitt lymphoma (BL) and high-grade B-cell lymphoma (HGBCL).

Supplemental figure S8. Expression of checkpoint molecules on memory CD8+ T cells from lymphoma samples. A. Representative flow cytometry plots and the percentage of PD-1+ CD8+ T_{mem} cells. **B.** Representative flow cytometry plots and the percentage of TIGIT+ CD8+ T_{mem} cells. **C.** Representative flow cytometry plots and the percentage of LAG-3+ CD8+ T_{mem} cells. **D.** Representative flow cytometry plots and the percentage of CD244+ CD8+ T_{mem} cells. **E.** Representative flow cytometry plots and the percentage of TIM3+ CD8+ T_{mem} cells. **F.** Representative flow cytometry plots and the percentage of CD160+ CD8+ T_{mem} cells. **G.** Representative flow cytometry plots and the percentage of PD-1+TIGIT+ CD8+ T_{mem} cells. **H.** Representative flow cytometry plots and the percentage of 41BB+ CD8+ T_{mem} cells. **I.** Representative flow cytometry plots and the percentage of OX40+ CD8+ T_{mem} cells. **J.** Representative flow cytometry plots and the percentage of ICOS+ CD8+ T_{mem} cells. The Mann-Whitney test was performed to compare statistical differences between rLT and NHL subtypes when comparing

percentages. Graphs show individual values and mean. (*P<0.05; **P<0.01; ***P<0.001; ****P<0.0001). Memory T cells (T_{mem}), reactive lymphoid tissue (rLT), follicular lymphoma (FL), diffuse large B-cell lymphoma (DLBCL), marginal zone lymphoma (MZL), mantle cell lymphoma (MCL), Burkitt lymphoma (BL) and high-grade B-cell lymphoma (HGBCL).

Supplemental figure S9. Expression of checkpoint molecules on memory CD4+ T cells from lymphoma samples. A. Representative flow cytometry plots and the percentage of PD-1+ CD4+ T_{mem} cells. **B.** Representative flow cytometry plots and the percentage of TIGIT+ CD4+ T_{mem} cells. **C.** Representative flow cytometry plots and the percentage of LAG-3+ CD4+ T_{mem} cells. **D.** Representative flow cytometry plots and the percentage of CD244+ CD4+ T_{mem} cells. **E.** Representative flow cytometry plots and the percentage of TIM3+ CD4+ T_{mem} cells. **F.** Representative flow cytometry plots and the percentage of CD160+ CD4+ T_{mem} cells. **G.** Representative flow cytometry plots and the percentage of PD-1+TIGIT+ CD4+ T_{mem} cells. **H.** Representative flow cytometry plots and the percentage of 41BB+ CD4+ T_{mem} cells. **I.** Representative flow cytometry plots and the percentage of OX40+ CD4+ T_{mem} cells. **J.** Representative flow cytometry plots and the percentage of ICOS+ CD4+ T_{mem} cells. The Mann-Whitney test was performed to compare statistical differences between rLT and NHL subtypes when comparing percentages. Graphs show individual values and mean. (*P<0.05; **P<0.01; ***P<0.001; ****P<0.0001). Memory T cells (T_{mem}), reactive lymphoid tissue (rLT), follicular lymphoma (FL), diffuse large B-cell lymphoma (DLBCL), marginal zone lymphoma (MZL), mantle cell lymphoma (MCL), Burkitt lymphoma (BL) and high-grade B-cell lymphoma (HGBCL).

Supplemental figure S10. Characterization of PD-1 expression on T cells. A. Representative flow cytometry plots, percentages and stacked bar graph of PD-1 high, low and negative expressions on CD8+ T_{mem} cells**. B.** Representative flow cytometry plots, percentages and stacked bar graph of PD-1 high, low and negative expressions on CD4+ T_{mem} cells. **C.** PD-1 expression on each differentiation subsets of CD8+ T cells. **D.** PD-1 expression on each differentiation subsets of CD4+ T cells. T_{mem}: memory T cells, negative (neg), reactive lymphoid tissue (rLT), follicular lymphoma (FL), diffuse large B-cell lymphoma (DLBCL), marginal zone lymphoma (MZL).

Supplemental figure S11. Expression of all analysed exhaustion markers on T cells in two dimensions. Representative tSNE plots showing the expression of PD-1, TIGIT, LAG-3, CD244, TIM3 and CD160 expressions on CD8+ and CD4+ T cells in rLT, FL and DLBCL. T-distributed stochastic neighbour embedding (tSNE), reactive lymphoid tissue (rLT), follicular lymphoma (FL), diffuse large B-cell lymphoma (DLBCL).

Supplemental figure S12. CD4+ T cell subpopulations. A. Representative flow cytometry plots of regulatory T-like (Treg-like) cells, defined as CD25+CD127low/- cells, and the percentage of Treg-like cells in rLT compared to FL, DLBCL and MZL. **B.** Representative gating strategy of T helper subpopulations out of CD4+ T cells and stacked bar plot of the main T helper subpopulations out of $CD4^+$ T cells including Th1 ($CXCR3^+CCR4^-CCR6^-$), Th2 ($CXCR3^-CCR4^+CCR6^-$), Th17 ($CCR4^+CCR6^+$). CCR10 expression was not evaluated due to the absence of a clearly distinguishable subpopulation of positive cells in the individuals assessed. The Mann-Whitney test was performed to compare statistical differences between rLT and NHL subtypes when comparing percentages. Graphs show individual values and mean. (*P<0.05; **P<0.01; ***P<0.001; ****P<0.0001). Reactive lymphoid tissue (rLT), follicular lymphoma (FL), diffuse large B-cell lymphoma (DLBCL), marginal zone lymphoma (MZL).

Supplemental figure S13. Correlation of the analysed parameters within each cell subpopulation. Correlogram depicting pairwise Pearson correlations among the analysed parameters, including the frequencies of the major lymphocyte subpopulations and the expression of markers within: **A.** malignant and non-malignant B cells, **B.** CD8+ T cells and **C**. CD4+ T cells. Dot colour and size represent the strength and direction of statistically significant correlations (P<0.05). White boxes indicate parameter pairs with non-significant correlations.

Supplemental figure S14. Correlation matrix of all the analysed TIM features. Correlogram depicting pairwise Pearson correlations among 54 immunophenotypic parameters analysed. Dot colour and size represent the strength and direction of statistically significant correlations (P<0.05). White boxes indicate parameter pairs with non-significant correlations.

Supplemental figure S15. TIM characteristics in NHL patients harbouring the most frequent mutations in our cohort. Log-transformed fold changes of the 54 immunophenotypic parameters comparing patients with mutations in *CREBBP* (n=28), *KMT2D* (n=27), *TNFRSF14* (n=14), *BTG1* (n=11) and *STAT6* (n=10) versus non-mutated patients. The Mann-Whitney test was performed to compare statistical differences. (*P<0.05; **P<0.01; ***P<0.001; ****P<0.0001).

Supplemental figure S16. TIM characteristics in FL patients harbouring *BCL2*-R. Log-transformed fold changes of the 54 immunophenotypic parameters comparing FL patients *BCL2*-R positive (n=23) versus *BCL2*-R negative (n=7) FL patients. The Mann-Whitney test was performed to compare statistical differences. (*P<0.05; **P<0.01; ***P<0.001; ****P<0.0001). *BCL2* rearrangement (*BCL2*-R).

Supplemental figure S17. Association of the TIM-based clustering and other clinical variables and prognostic factors. A. Stacked bar plot describing the status of the germinal centre structure of FL patients from C1 and C2 clusters. **B.** Multivariate analysis of TTT including the TIM-based clustering, FLIPI score, tumour burden and BCL-2-R status. **C.** Multivariate analysis of PFS including the TIM-based clustering, FLIPI score, tumour burden and BCL-2-R status. Germinal centre (GC), time-to-treatment (TTT), progression-free survival (PFS), Follicular Lymphoma International Prognostic Index (FLIPI), *BCL2* rearrangement (*BCL2*-R).

Supplemental figure S1

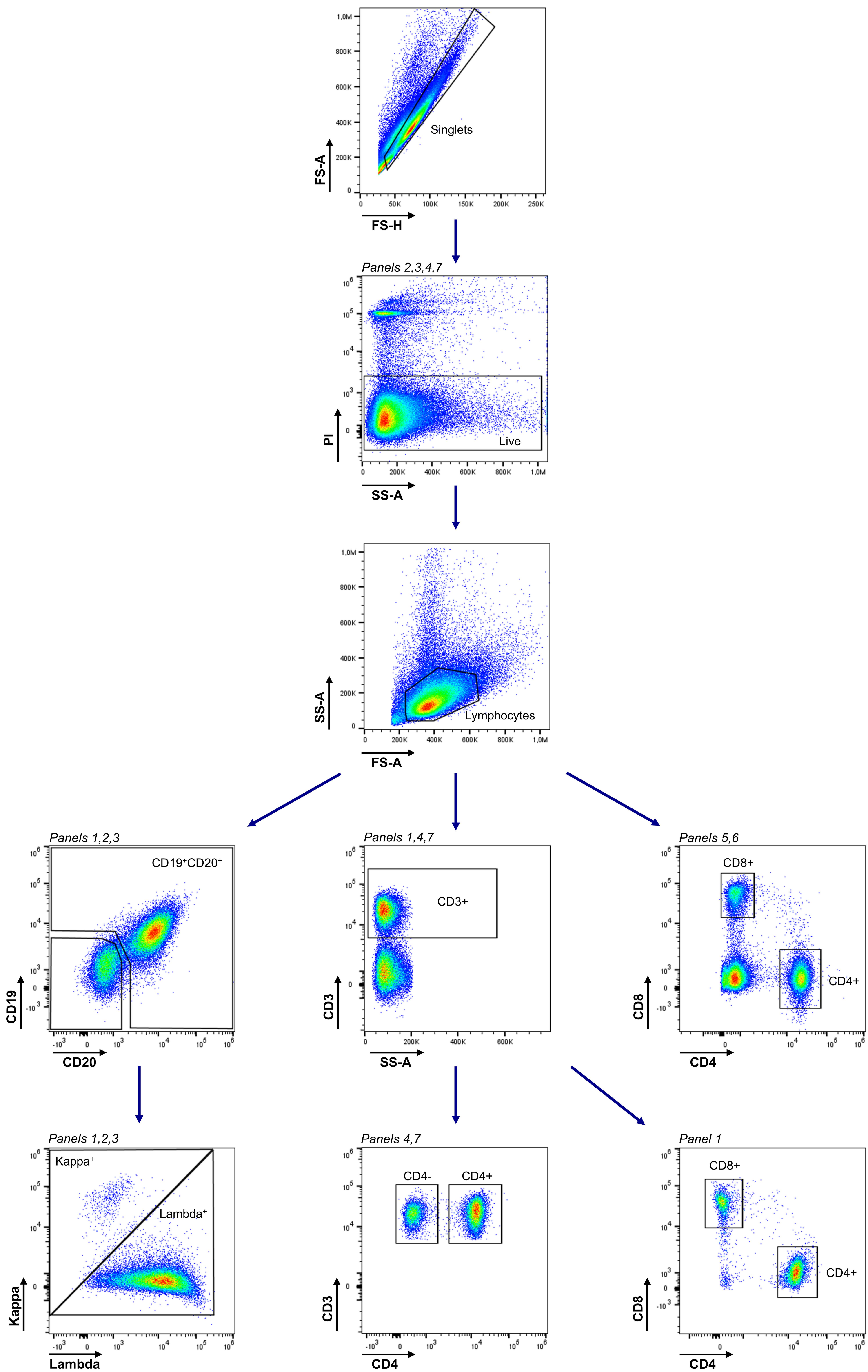

Supplemental figure S2

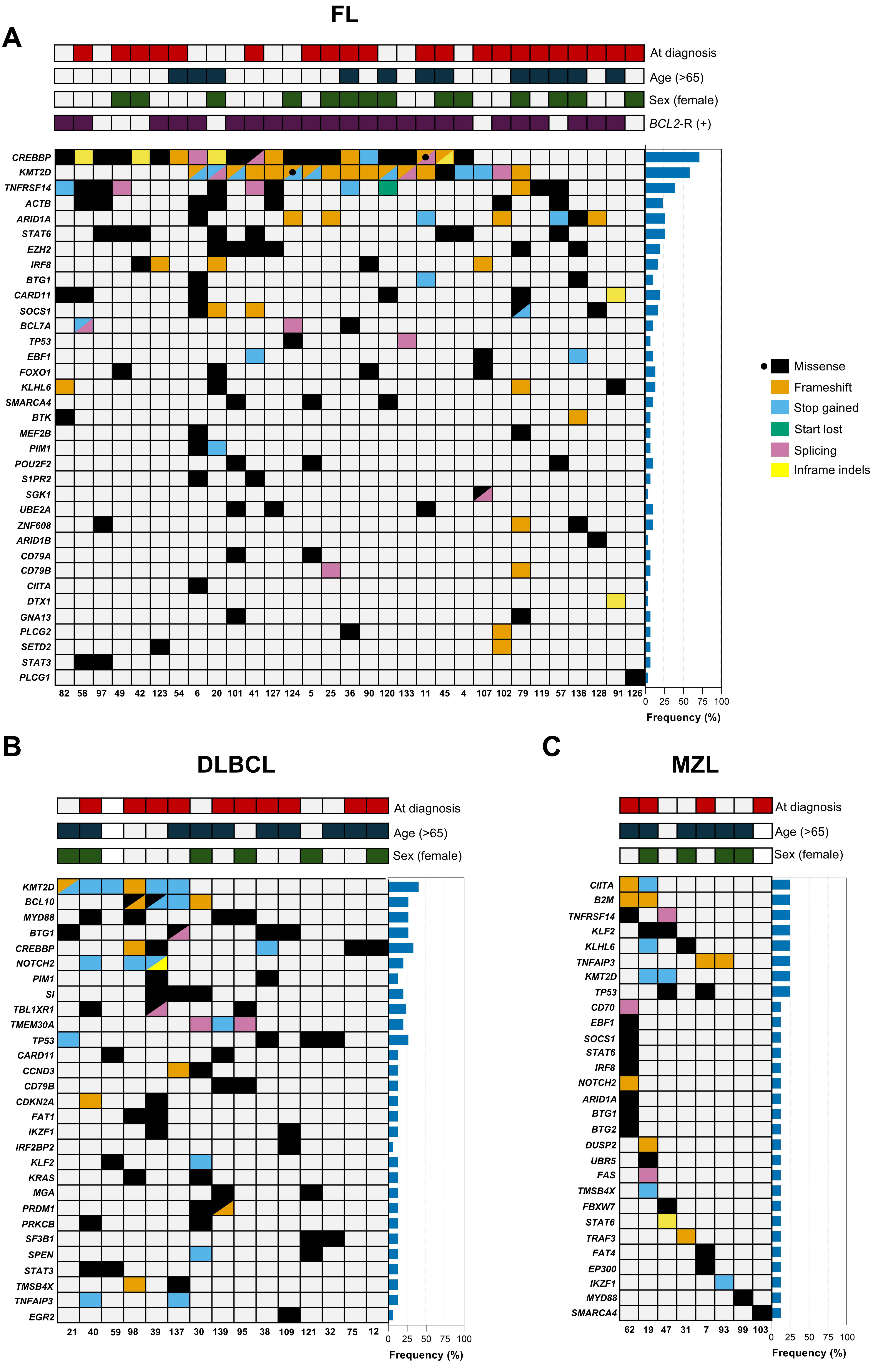

Supplemental figure S3

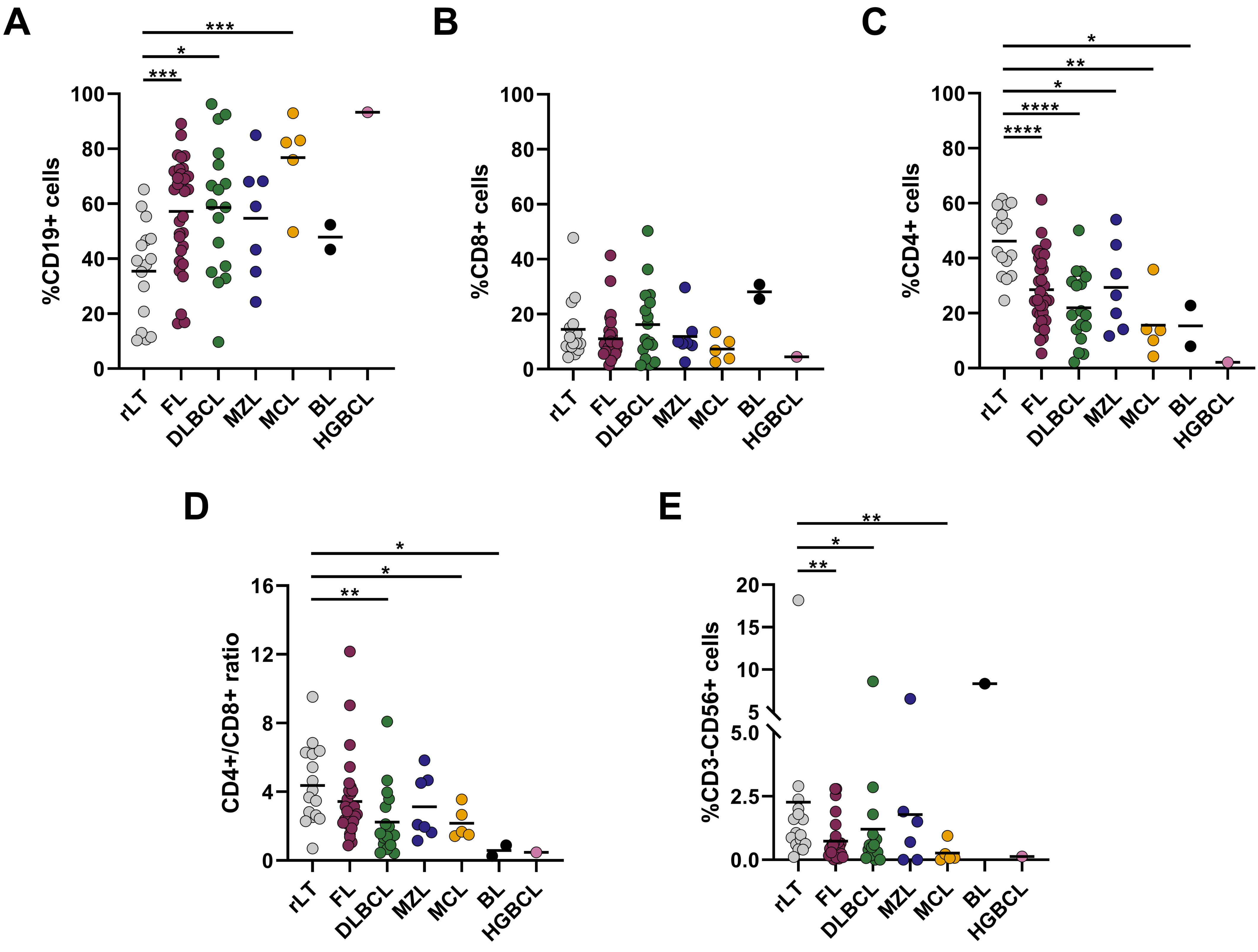

Supplemental figure S4

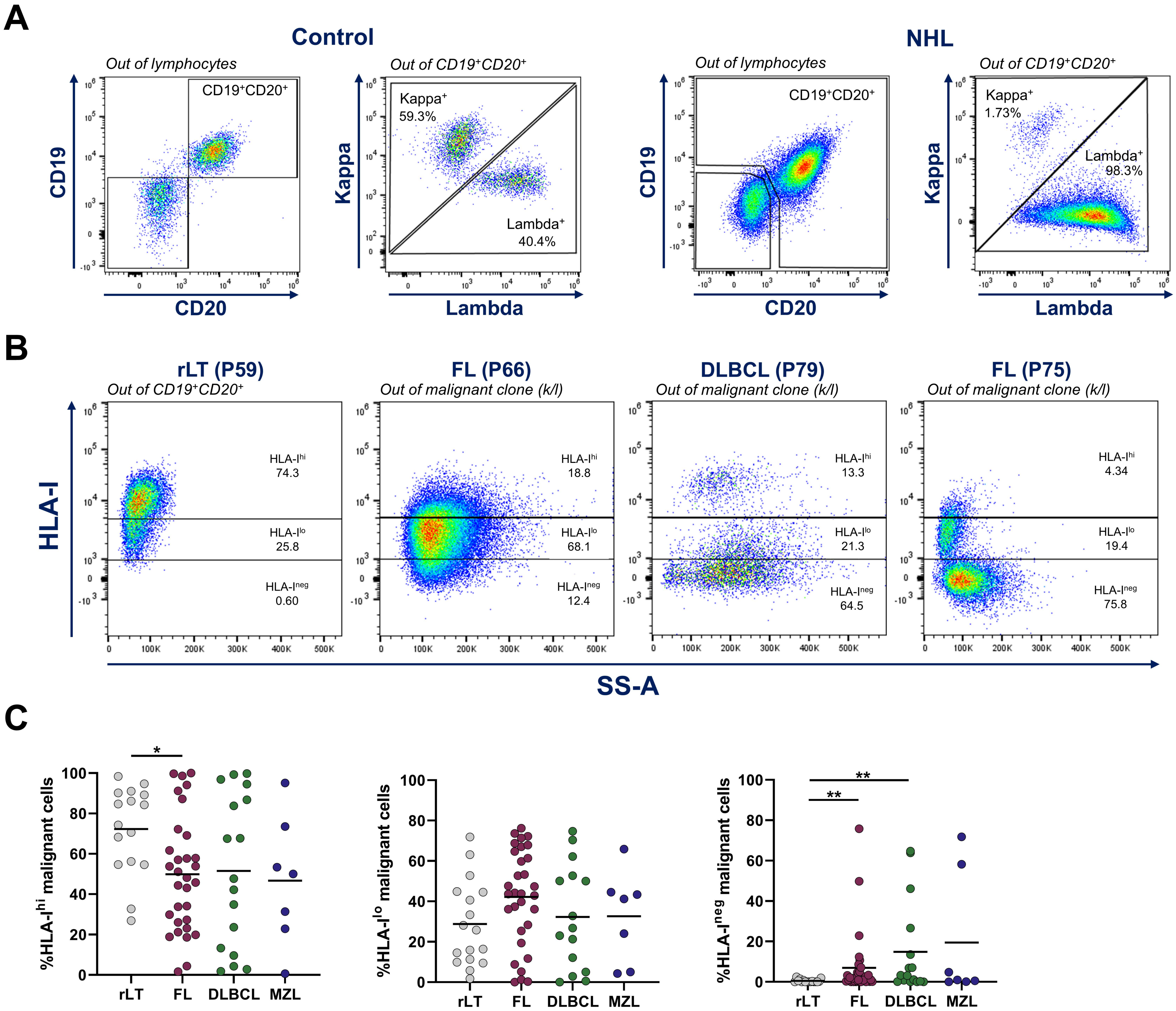

Supplemental figure S5

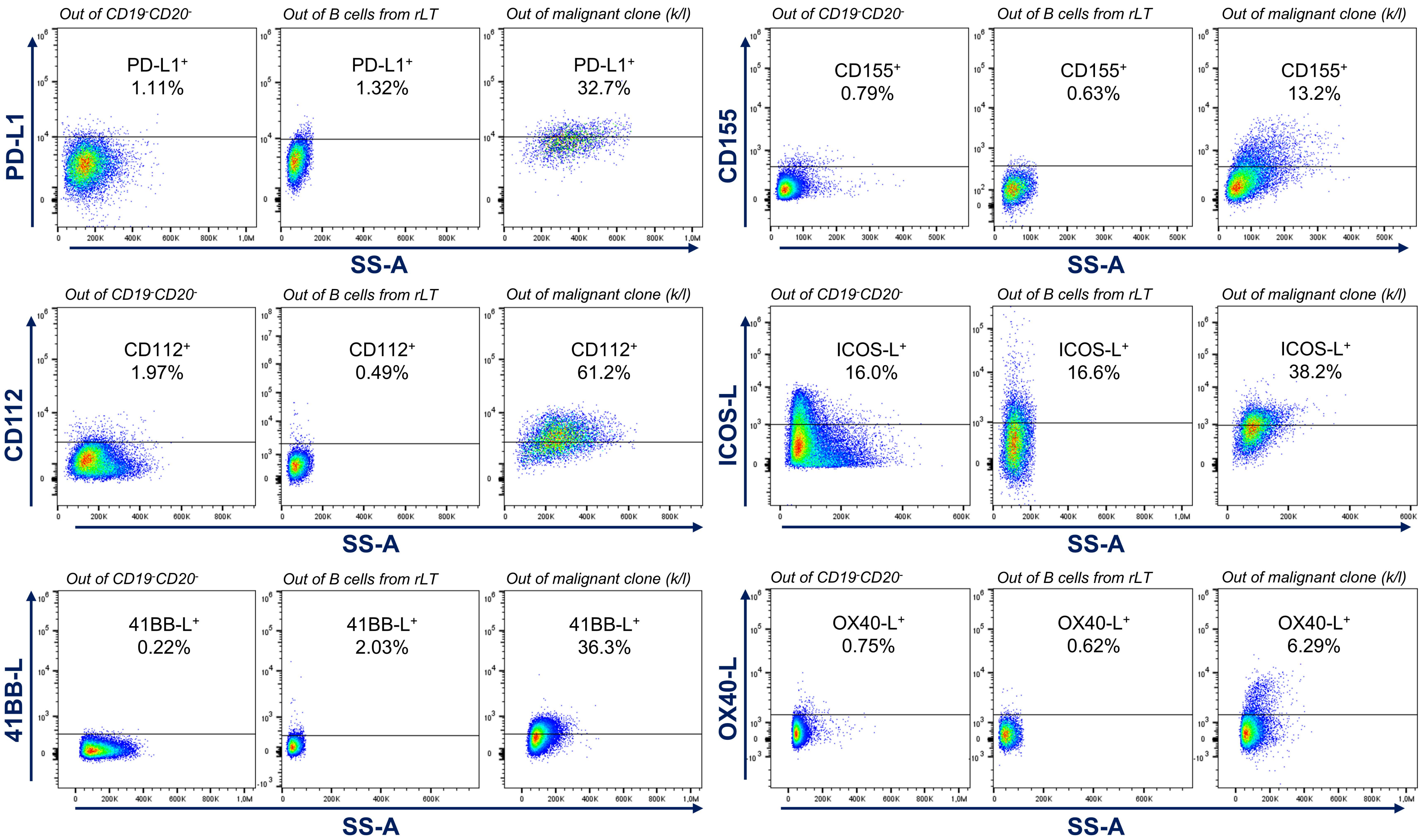

Supplemental figure S6

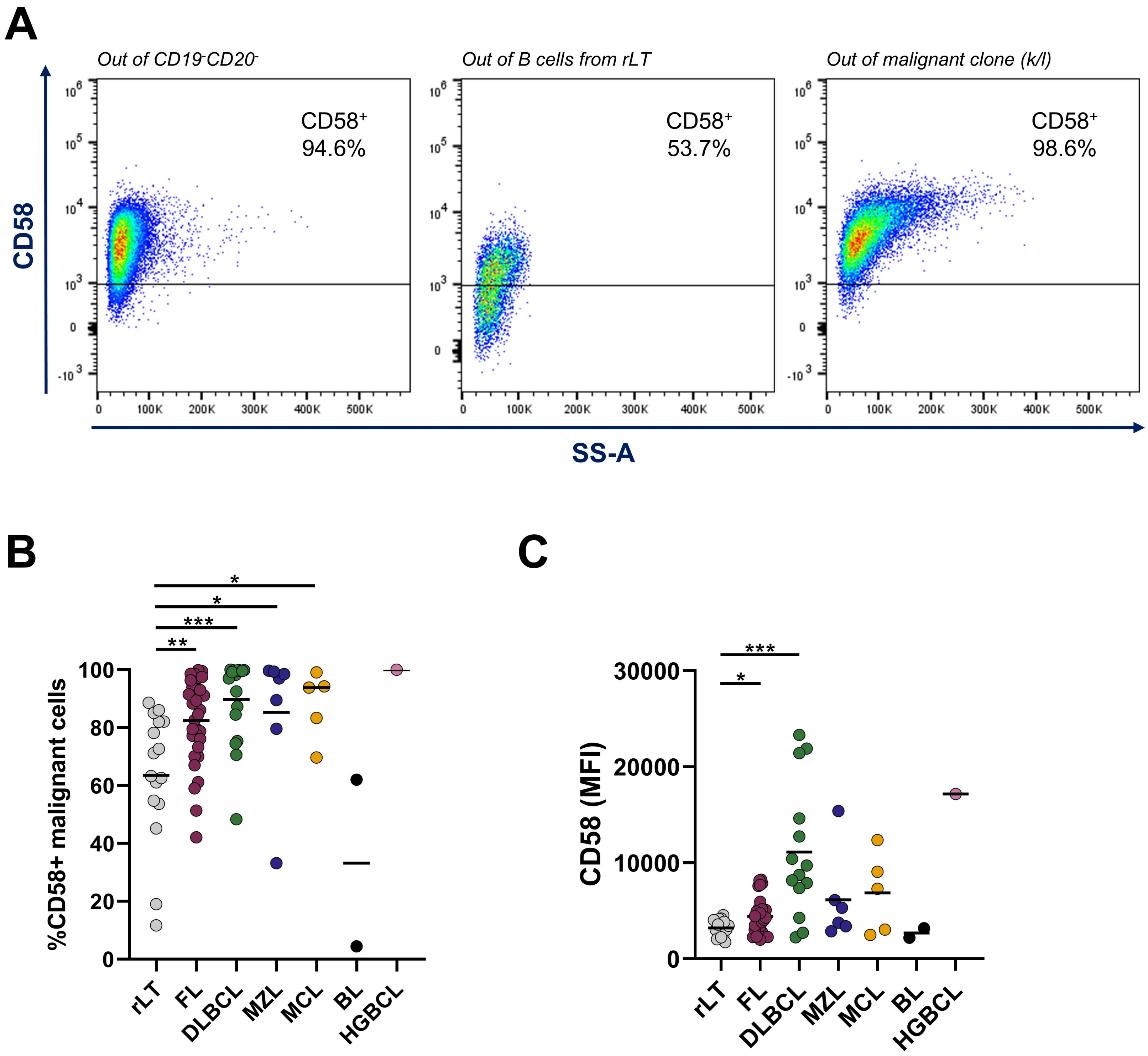

Supplemental figure S7

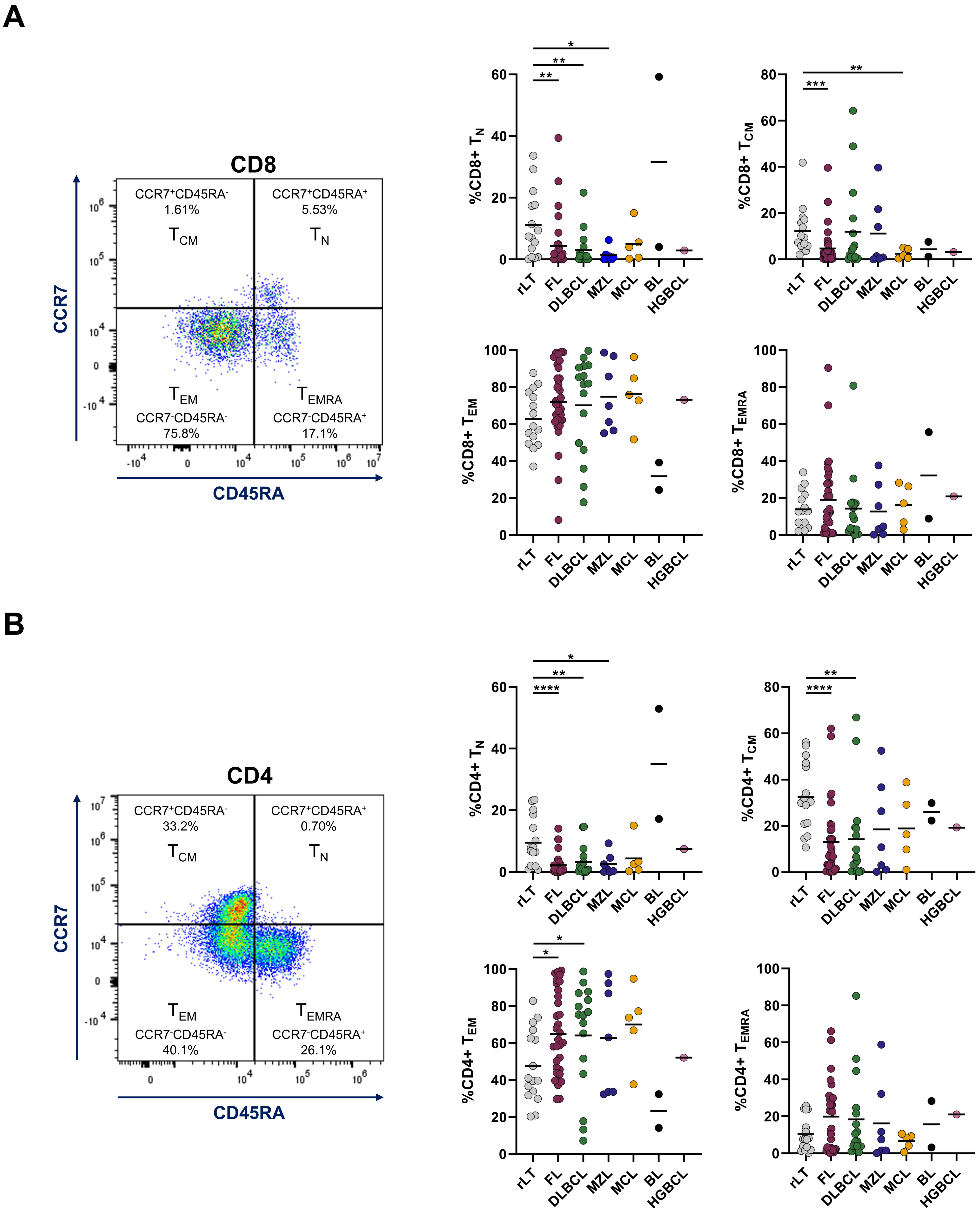

Supplemental figure S8

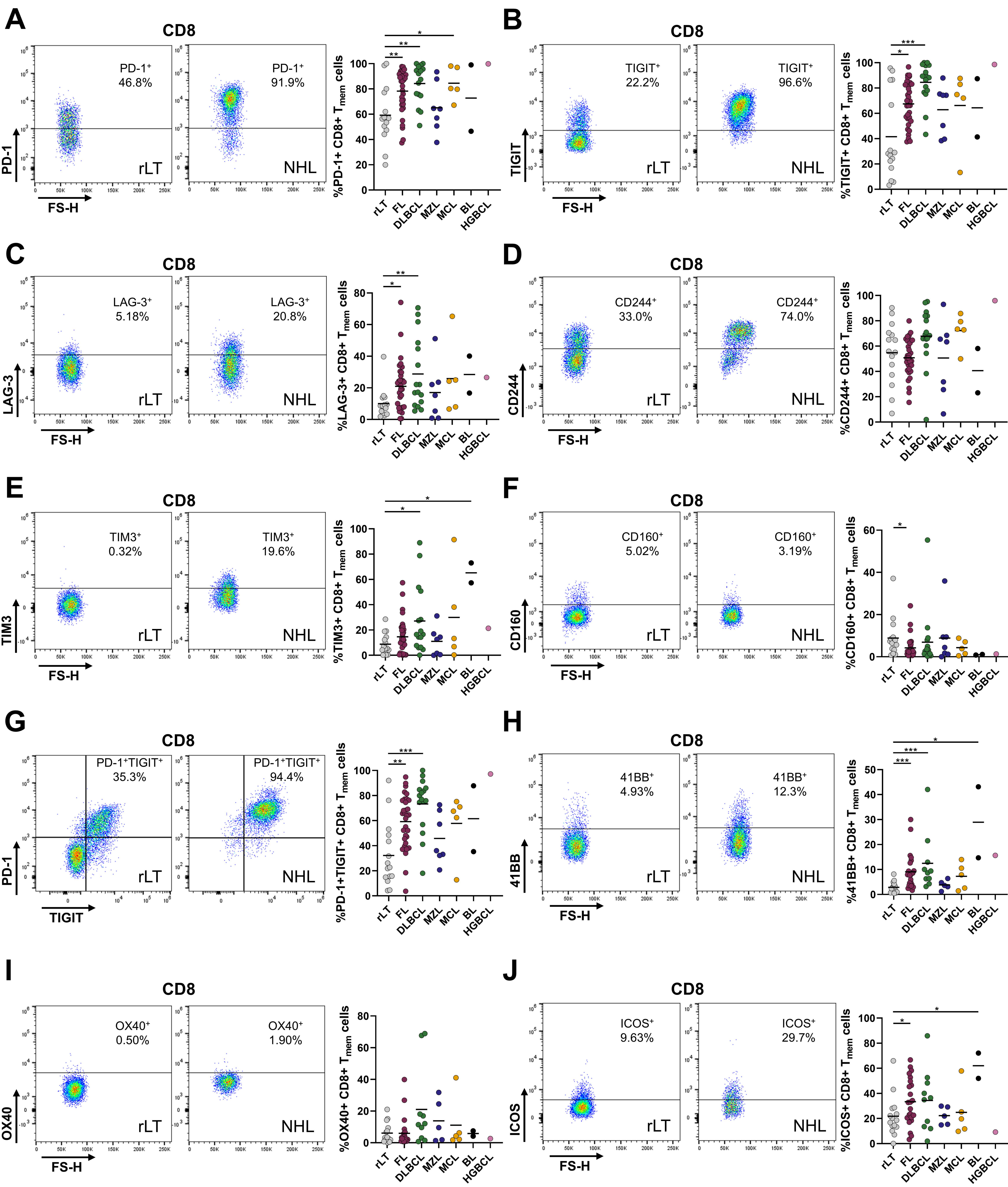

Supplemental figure S9

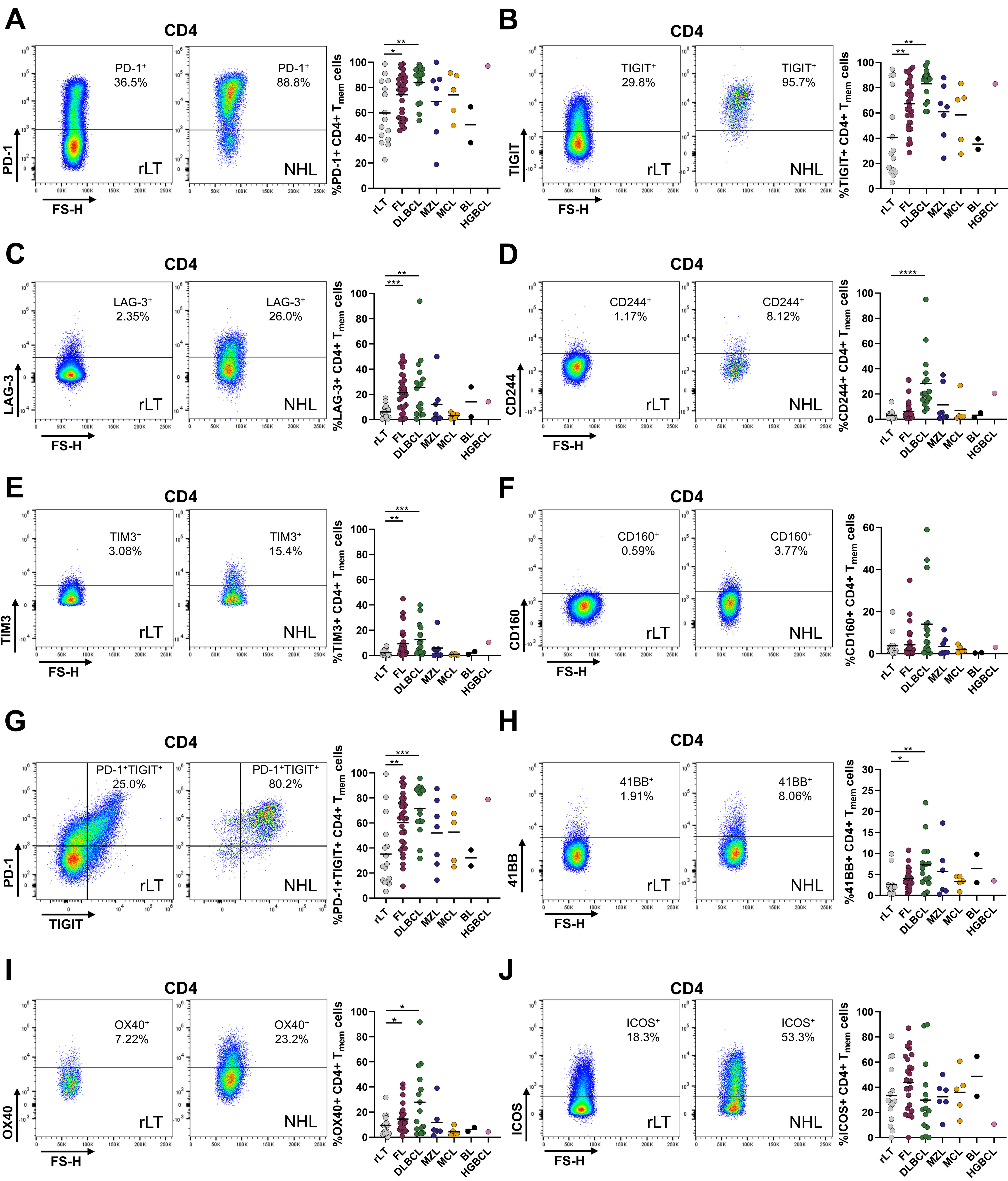

Supplemental figure S10

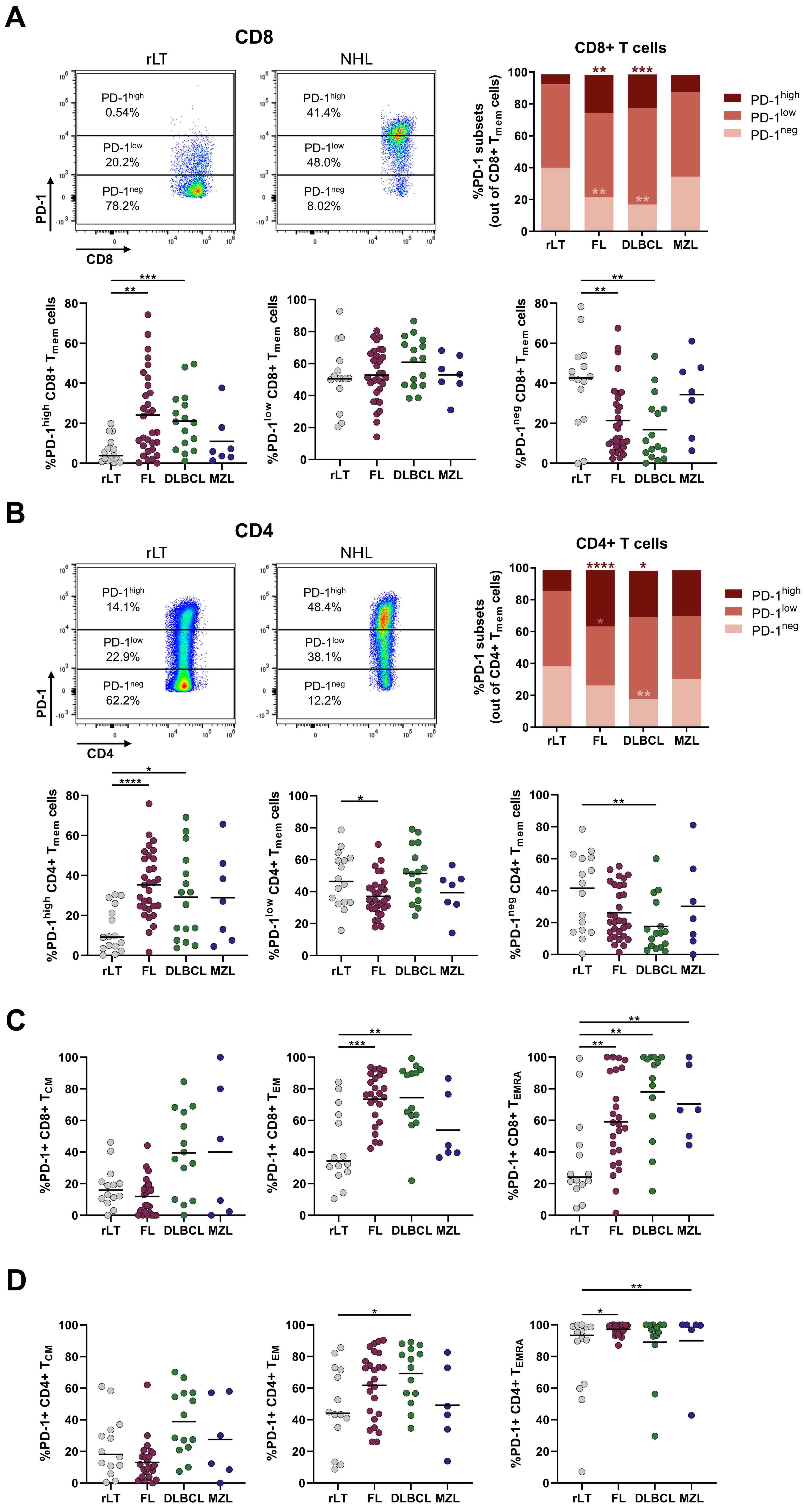

Supplemental figure S11

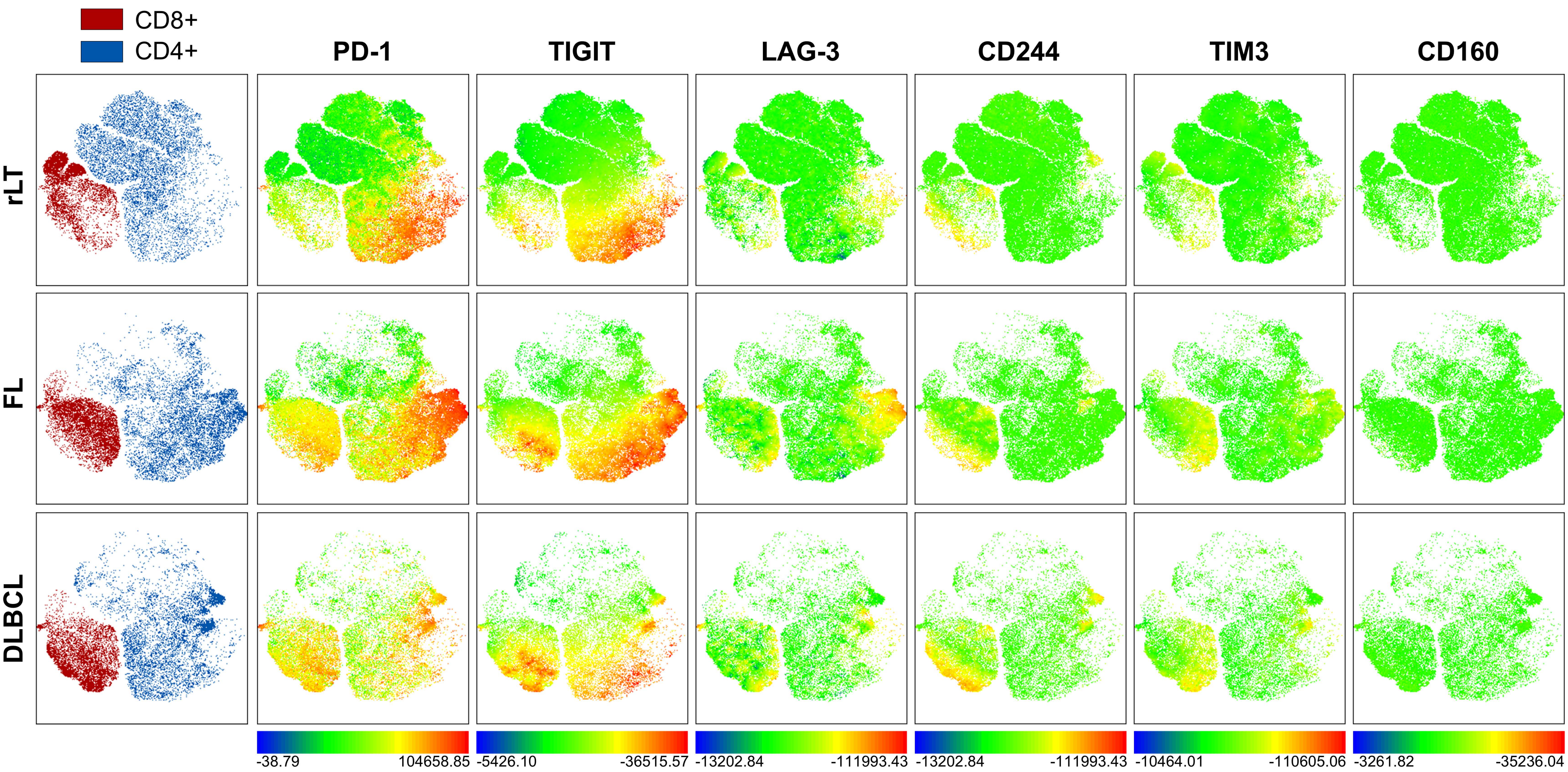

Supplemental figure S12

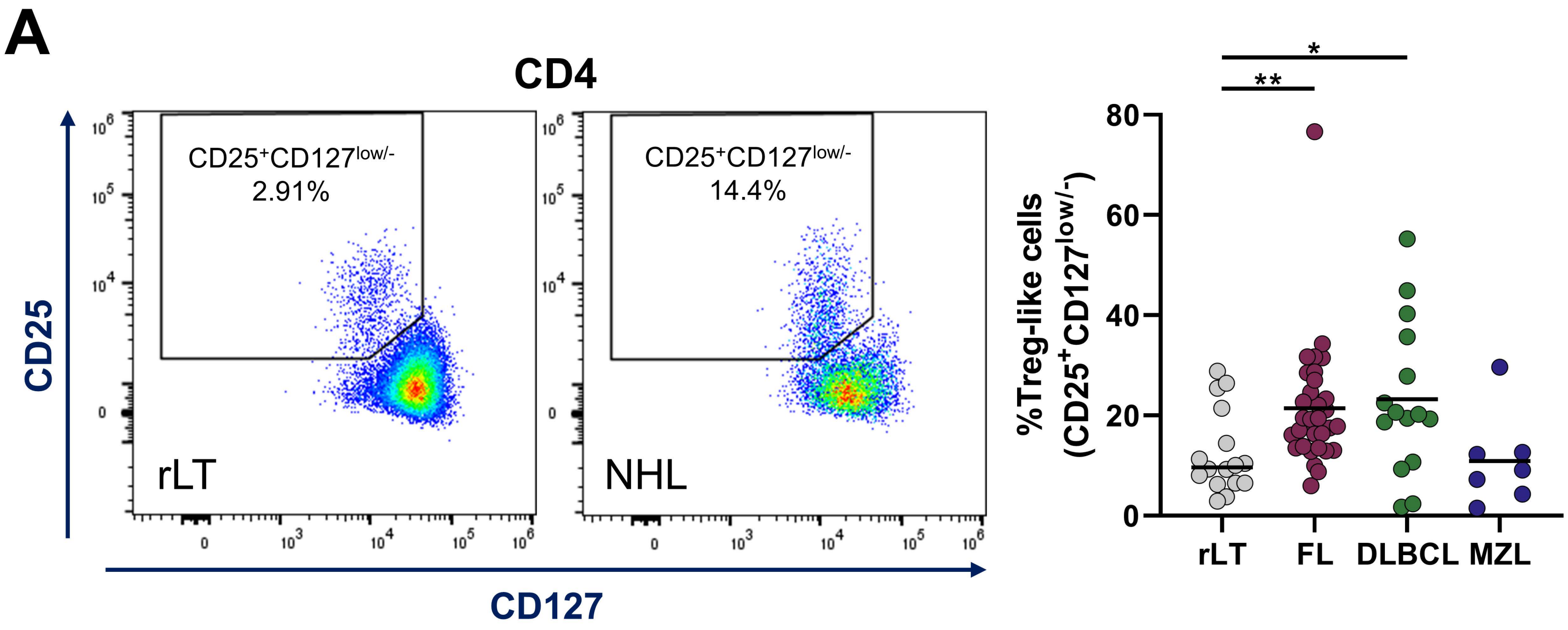

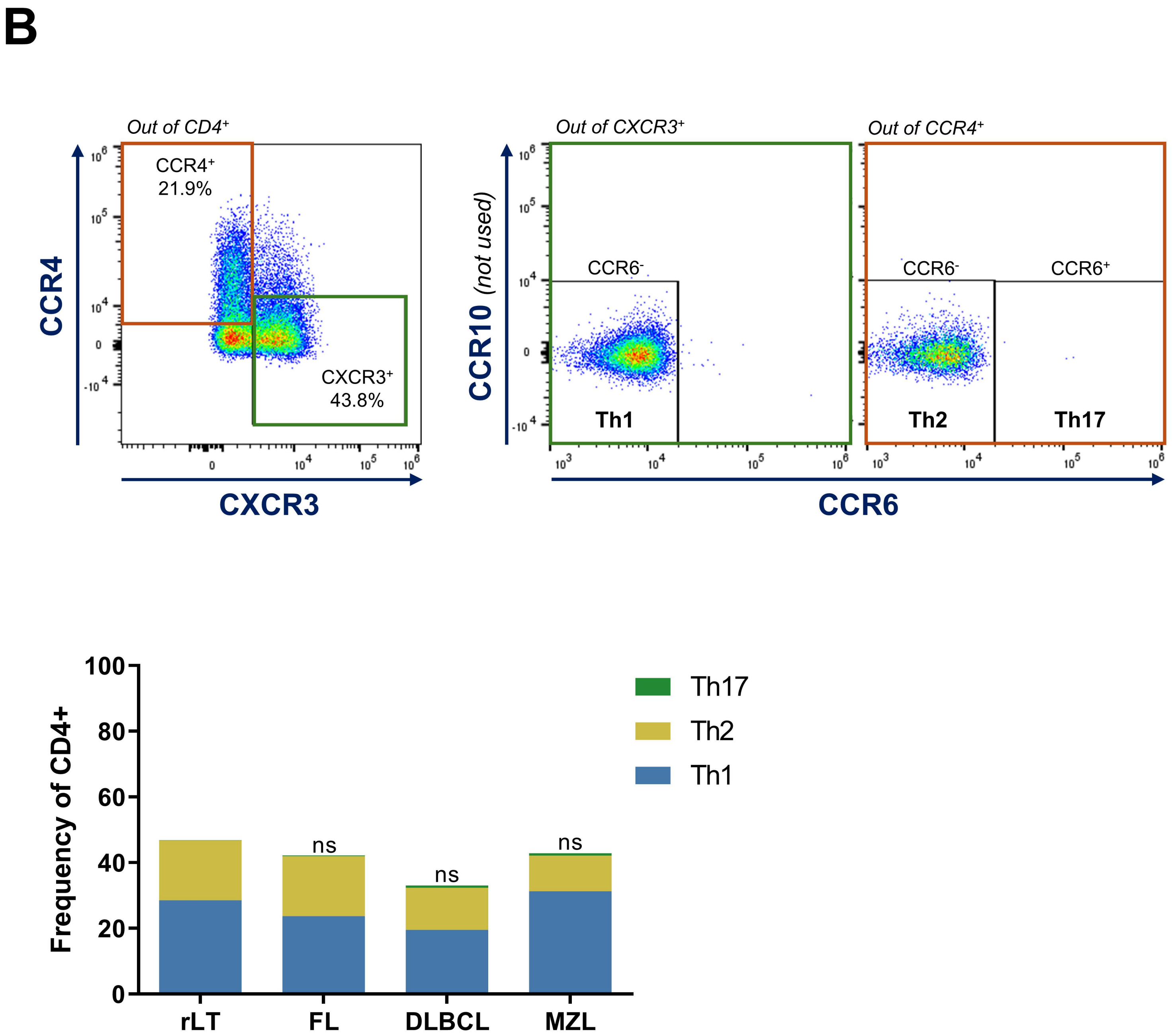

Supplemental figure S13

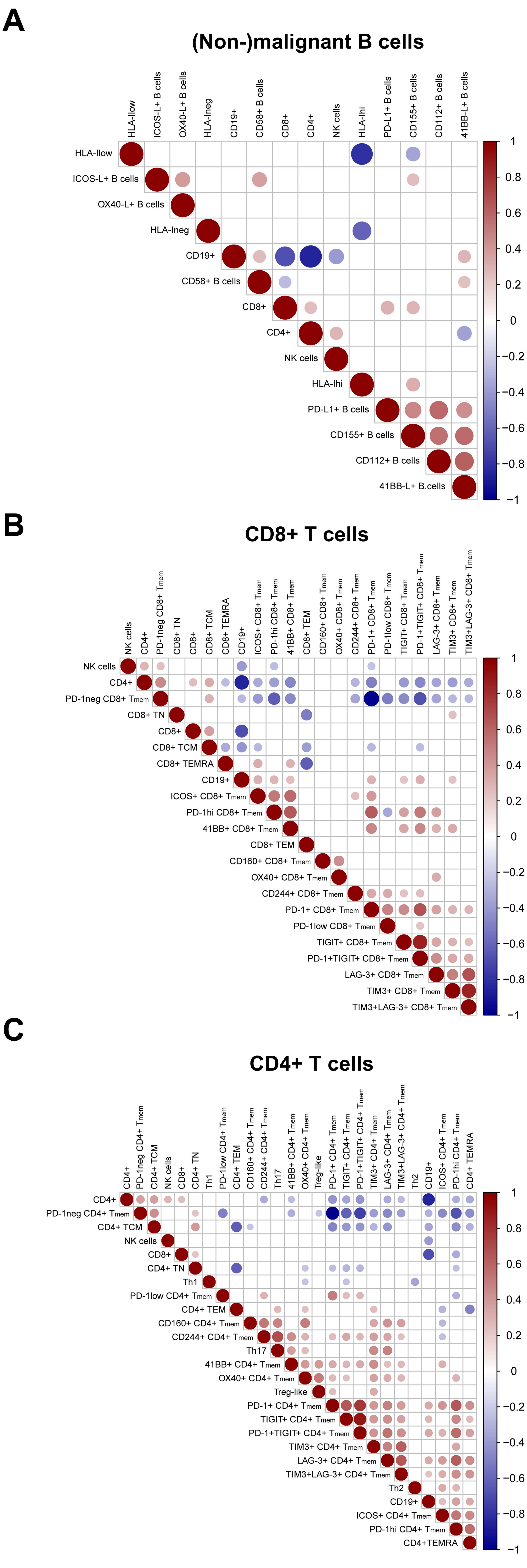

Supplemental figure S14

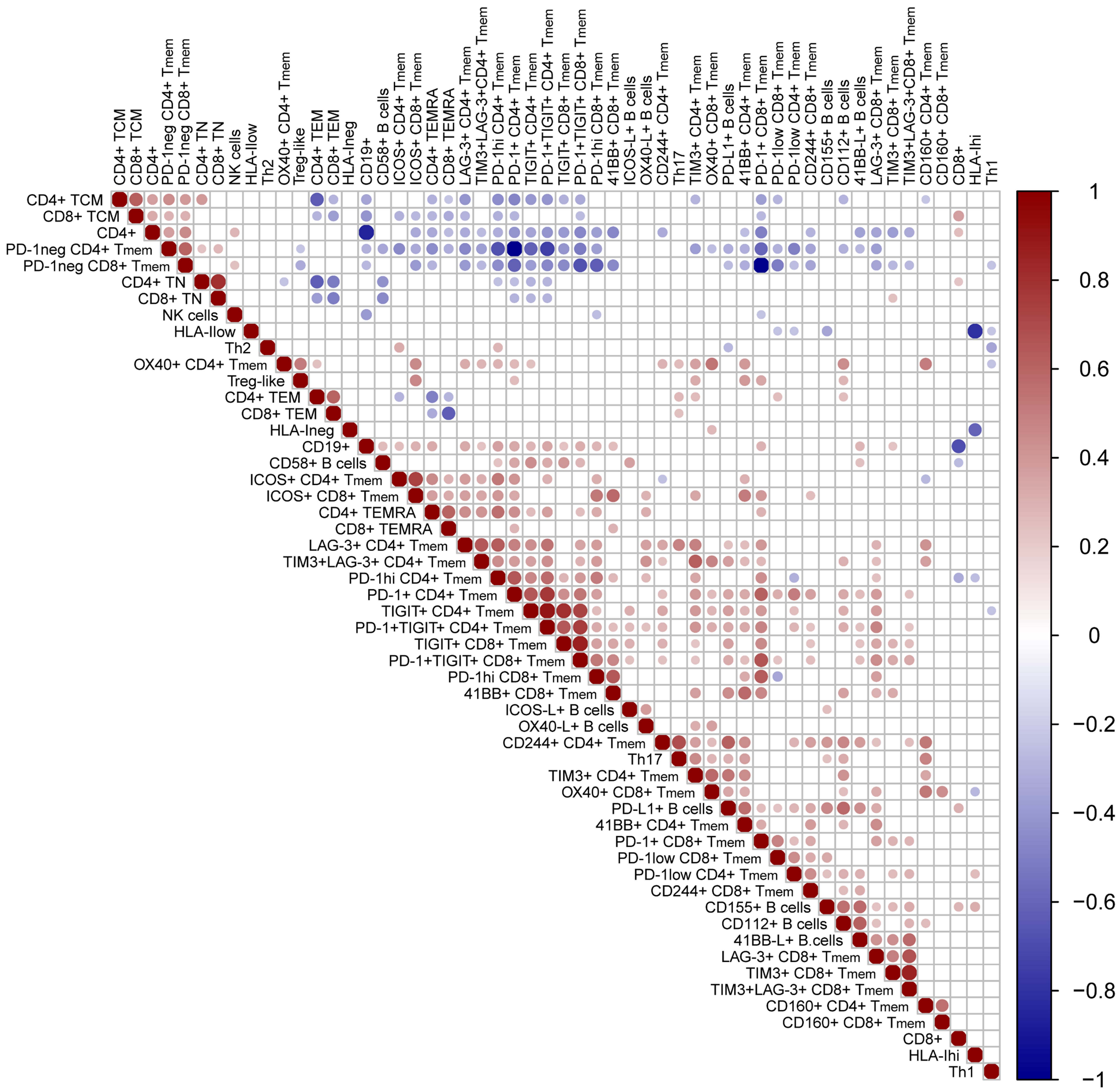

Supplemental figure S15

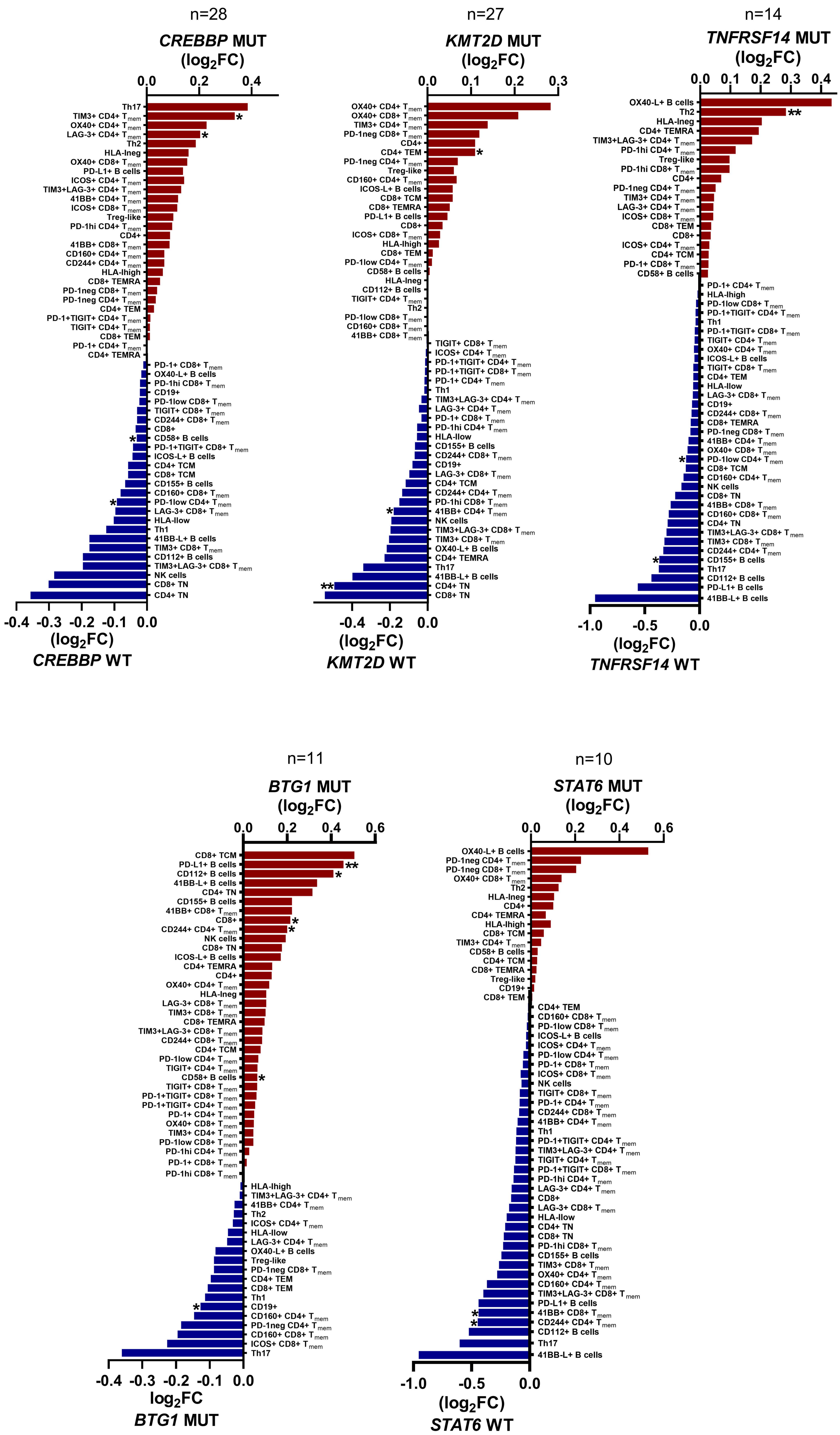

Supplemental figure S16

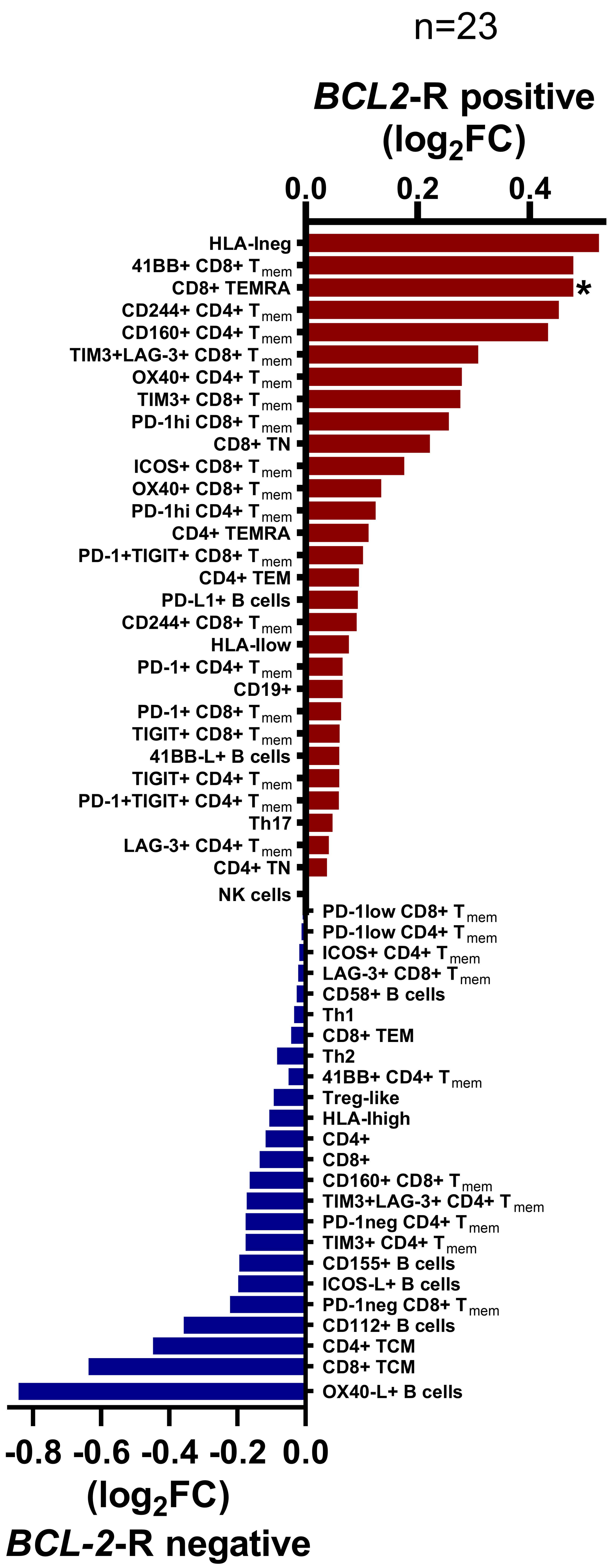

Supplemental figure S17

A

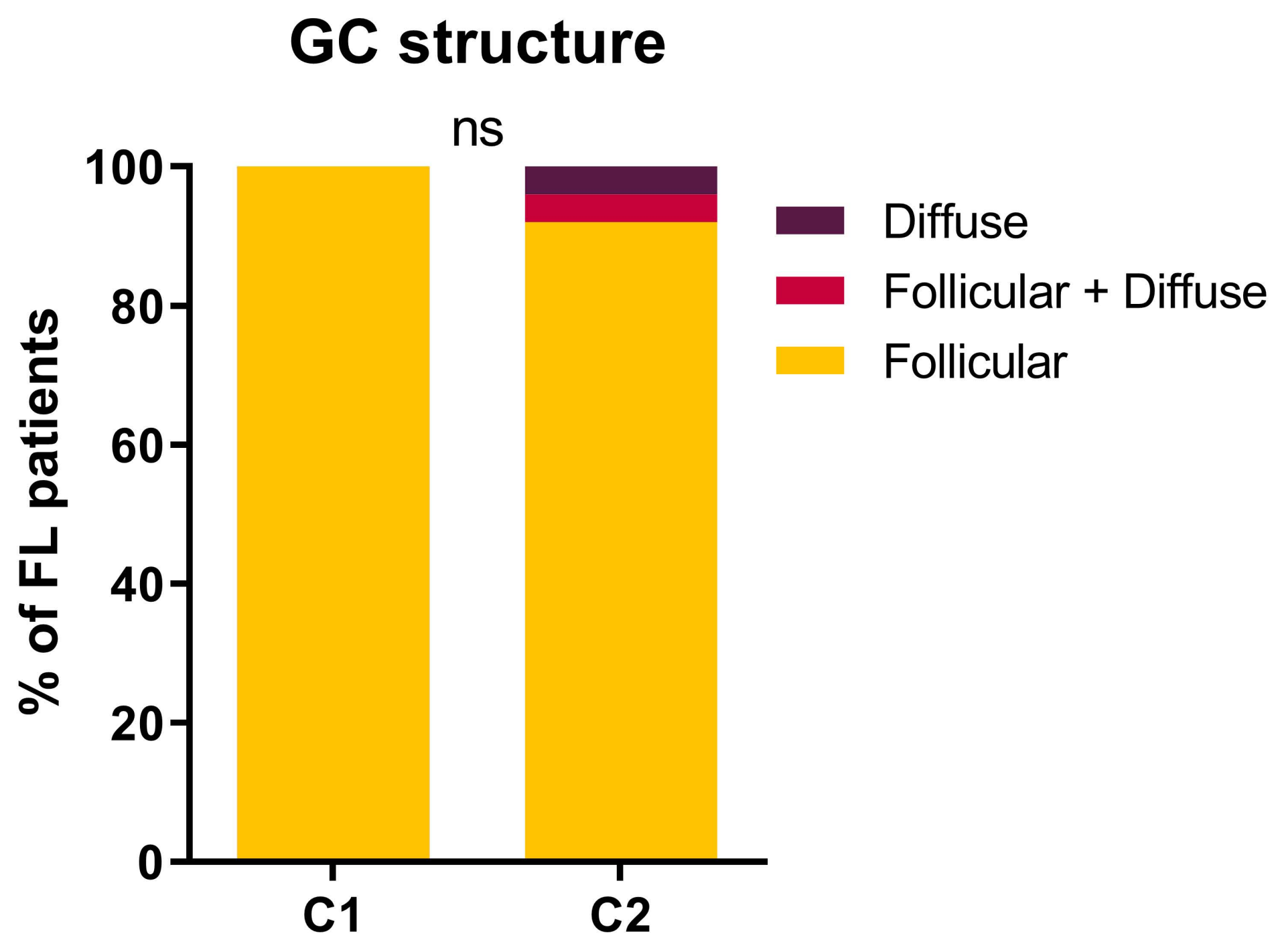

B

TTT

Variable		N	Hazard ratio		p
**Cluster**	C1	6		Reference	
	C2	23		1.70 (0.39, 7.46)	0.480
**FLIPI**	0–2	14		Reference	
	3–5	15		5.44 (1.60, 18.46)	0.007
**Tumor Burden**	low	20		Reference	
	high	9		1.46 (0.55, 3.85)	0.444
**BCL2-R**	No	7		Reference	
	Yes	22		0.57 (0.17, 1.90)	0.362

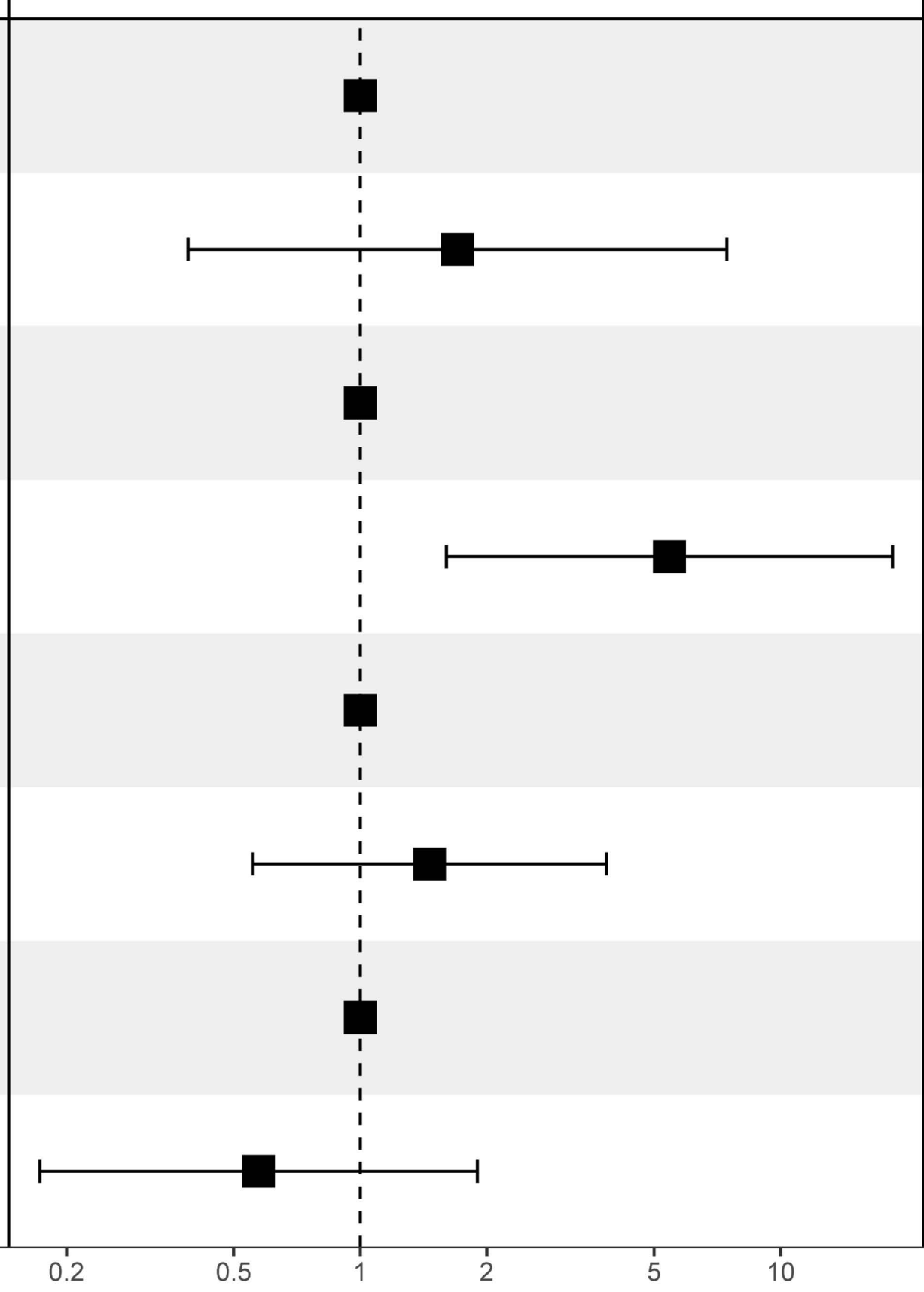

C

PFS

Variable		N	Hazard ratio		p
**Cluster**	C1	7		Reference	
	C2	22		0.75 (0.04, 13.06)	0.841
**FLIPI**	0–2	15		Reference	
	3–5	14		4.72 (0.56, 39.62)	0.153
**Tumor Burden**	low	20		Reference	
	high	9		1.96 (0.55, 7.06)	0.302
**BCL2-R**	No	7		Reference	
	Yes	22		2.54 (0.26, 24.67)	0.422

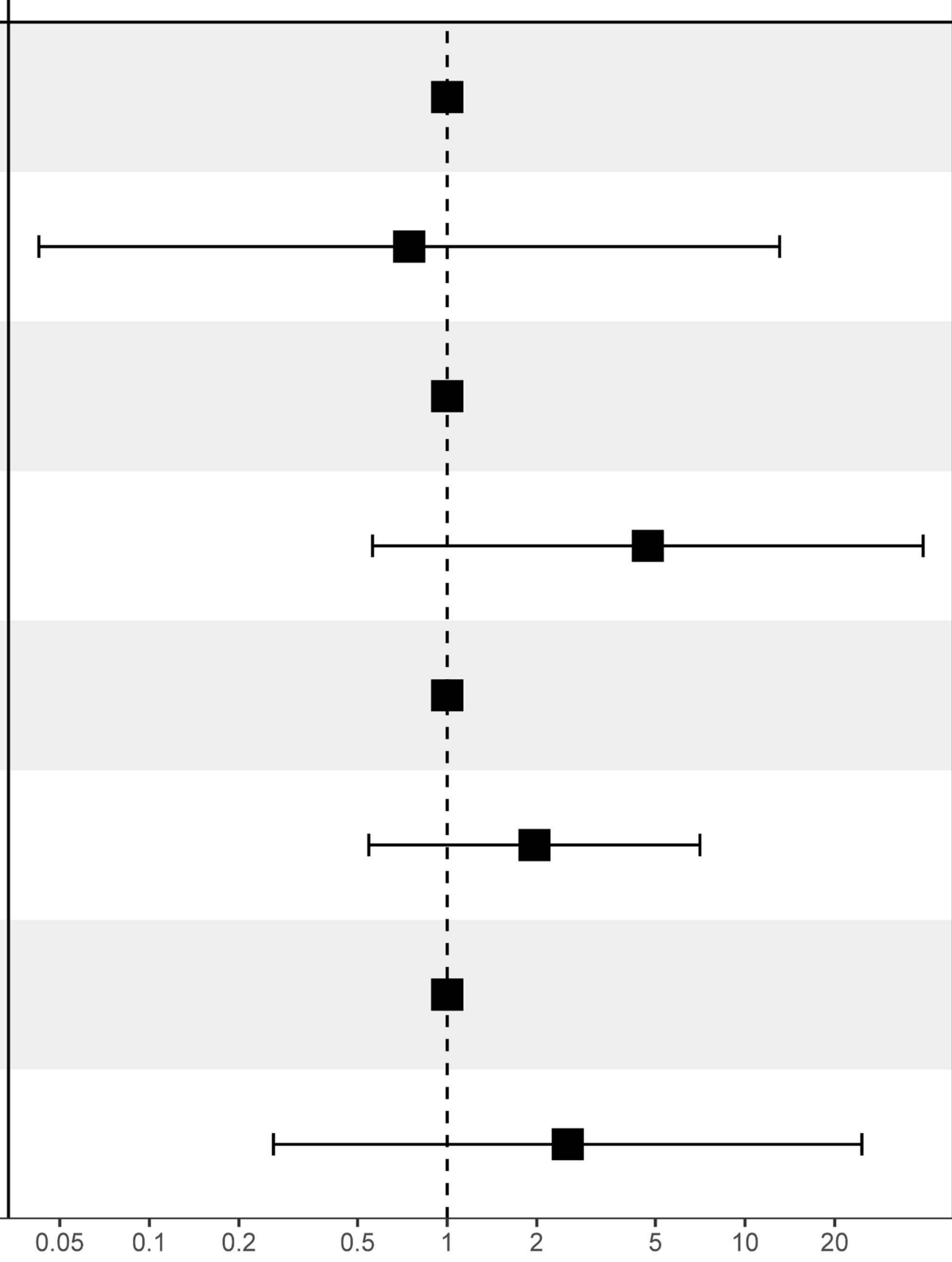

Supplement: Supplementary file 1 [file DataSheet1.pdf]
